# Magnetic Resonance Imaging Assessment of Abdominal Ectopic Fat Deposition in Correlation With Cardiometabolic Risk Factors

**DOI:** 10.3389/fendo.2022.820023

**Published:** 2022-03-30

**Authors:** Qin-He Zhang, Lu-Han Xie, Hao-Nan Zhang, Jing-Hong Liu, Ying Zhao, Li-Hua Chen, Ye Ju, An-Liang Chen, Nan Wang, Qing-Wei Song, Li-Zhi Xie, Ai-Lian Liu

**Affiliations:** ^1^Department of Radiology, The First Affiliated Hospital of Dalian Medical University, Dalian, China; ^2^Department of Pathology and Forensics, Dalian Medical University, Dalian, China; ^3^MR Research, GE Healthcare, Beijing, China

**Keywords:** ectopic fat deposition, cardiometabolic risk factors, abdominal fat distribution, magnetic resonance imaging, obesity

## Abstract

**Purpose:**

Ectopic fat accumulation and abdominal fat distribution may have different cardiometabolic risk profiles. This study aimed to assess the associations between various magnetic resonance imaging (MRI)-acquired fat depots and cardiometabolic risk factors.

**Methods:**

A total of 320 subjects with median age of 59 years, 148 men and 172 women, were enrolled in the study. Visceral adipose tissue (VAT) area and fat fraction (FF), subcutaneous adipose tissue (SAT) area and FF at the L1–L2 levels, preperitoneal adipose tissue (pPAT) area and FF, hepatic FF, pancreatic FF, and intramuscular FF were assessed by MRI FF maps. The associations of various MRI-acquired fat depots with blood pressure, glucose, and lipid were examined using sex-stratified linear regression. Logistic regression stratified by sex was used to analyze the association of various MRI-acquired fat depots with the risk of hypertension, T2DM, and dyslipidemia.

**Results:**

The intraclass correlation coefficient (ICC) values were >0.9, which suggested good interobserver and intraobserver agreement. VAT area, V/S, hepatic fat, pancreatic fat, and pPAT rather than SAT area were significantly associated with multiple cardiometabolic risk factors (all *p* < 0.05). However, the patterns of these correlations varied by sex and specific risk factors. Also, VAT and SAT FF were only significantly associated with multiple cardiometabolic risk factors in women (all *p* < 0.05).

**Conclusions:**

VAT, hepatic fat, pancreatic fat, and pPAT were associated with cardiovascular metabolic risk factors independent of BMI. The patterns of these correlations were related to gender. These findings further the understanding of the association between ectopic fat deposition and cardiometabolic risk factors and help to better understand the obesity heterogeneity.

## 1 Introduction

Obesity is becoming one of the most significant public health problems and a leading cause of preventable death. Fatty acids produced in the human body are predominantly stored in adipose tissue in the form of triglycerides (TG). As the primary site of excessive TG storage, subcutaneous adipose tissue (SAT) capacity is limited. When SAT cannot store excessive amounts of energy, excessive TG leads to the accumulation of triglycerides in visceral adipose tissue (VAT), preperitoneal adipose tissue (pPAT), and nonadipose tissues, such as the heart, liver, pancreas, and skeletal muscles. These fat depots are described as “ectopic fat depots” (1, 2), which have been identified as an essential diagnostic and prognostic marker for the onset, progression, and mortality risk of cardiometabolic disease (1, 3–9). Obesity is a heterogeneous condition, and some obese individuals can be metabolically healthy (10). Thus, specific patterns of body fat deposition may confer different cardiometabolic risks (11).

Previously, the assessment of abdominal fat distribution mainly relied on several anthropometric measures, e.g., body mass index (BMI), waist-to-hip ratio (WHR), or waist circumference (WC), and/or broadly available clinical tools, such as dual-energy x-ray absorptiometry (DXA) and bioelectrical impedance analysis (BIA) (12–15). Still, none of these approaches mentioned above can evaluate the regional fat distribution and ectopic fat deposition. By contrast, computed tomography (CT) and magnetic resonance imaging (MRI) can be used to visually evaluate the abdominal fat distribution and ectopic fat deposition. Particularly, they are noninvasive, fast, and accurate, making them an ideal clinical indicator for fat quantification and monitoring changes in visceral and ectopic fat over time. The MRI protocol for the iterative decomposition of water and fat with echo asymmetry and least-squares estimation-iron quantification (IDEAL-IQ) sequence is a new method to fat quantification and can lead to a more accurate measurement of fat content because of a low flip angle for suppressing the longitudinal relaxation effects, and multiecho acquisition permit correction of the transverse relaxation effects. It has also been widely used to assess the fat quantification of different tissues (16).

Cardiometabolic risk factors included hypertension, type 2 diabetes mellitus (T2DM), dyslipidemia, and others. The simultaneous coexistence of two or more risk factors in a person at the same time has been recognized as clustering of cardiometabolic risk factors (CCRFs) (17), while the synergy of CCRFs can increase the risk of cardiovascular disease. In the early stages of cardiometabolic risk factors, deleterious and progressive changes in each organ are often asymptomatic and possibly reversible. Thus, a lifestyle modification can reduce morbidity and mortality of cardiometabolic risk factor-related diseases (18, 19). Defining imaging markers of high-risk fat accumulation might have important implications for prediction of cardiometabolic risk factors and early prevention or therapeutic intervention. Currently, studies that performed a direct comparison between various MRI-acquired fat depots accumulated in six abdominal regions (VAT, SAT, pPAT, liver, pancreas, and muscle) and multiple cardiometabolic risk factors are lacking. Accordingly, it remains unknown which MRI-acquired fat depots contribute the most to the individual cardiometabolic risk factors.

The aim of the present study was to assess the association between the various MRI-acquired fat depots and cardiometabolic risk factors. We hypothesized that the association between each fat content and cardiometabolic risk factors varies with the type of fat depots; there are fat depots that are more strongly associated with cardiometabolic risk factors than others, and they may therefore serve as imaging biomarkers in prediction, early prevention, or therapeutic intervention of cardiometabolic risk factors.

## 2 Materials and Methods

### 2.1 Study Population

This single-center, retrospective study included 4,718 patients who underwent upper abdominal MRI examination between January 2017 and August 2020. The target population was the general population. Of these participants, those who met at least one of the following criteria were excluded: age <18 years (*n* = 9); a history of heavy drinking (alcohol consumption ≥30 g/week in men or ≥20 g/week in women in the last 10 years) (*n* = 78); evidence of cirrhosis, malignant liver tumor, large benign liver tumor, liver posthepatectomy, and decompensated liver diseases (*n* = 992); evidence of other liver diseases (*n* = 171), including viral hepatitis, autoimmune liver diseases, drug-induced liver injury, etc.; evidence of pancreas diseases (*n* = 840), including acute or chronic pancreatitis, autoimmune pancreas diseases, pancreas tumor, pancreas postpancreatectomy, pancreatic trauma; intrahepatic bile, or pancreatic duct dilation (*n* = 126); thyroid diseases (*n* = 62); evidence of ascites, mesenteric injuries, huge abdominal mass, abdominal wall edema, and postostomy (*n* = 31); radiotherapy, chemotherapy, immunosuppressive therapy, antiviral therapy, and endocrine therapy (*n* = 1213); pregnancy (*n* = 3); suspected secondary hypertension and other types of diabetes except T2DM (*n* = 11); missing cardiometabolic risk factors (*n* = 858); missing IDEAL-IQ sequence (*n* = 130); missing covariates (*n* = 2); weight change by more than 5% (within 1 month) (*n* = 185); poor image quality (poor signal-to-noise ratio or motion artifacts) (*n* = 8). Finally, a total of 320 subjects (148 men and 172 women) were included in the analysis.

This retrospective study was approved by review board of the First Affiliated Hospital of Dalian Medical University, and a waiver of informed consent was remitted.

### 2.2 MRI Examinations

In this study, the MRI scanner (GE Medical Systems, Inc., Waukesha, WI, USA) with an eight-channel phased-array body coil was used. The patients fasted for 4–6 h and were trained to exhale and hold their breath for more than 20 s before scanning. The subjects were placed in the supine position during examination. A three-plane localization imaging gradient-echo sequence was performed at the beginning of acquisition.

IDEAL-IQ sequence and routine MRI Ax T1 FSPGR, Ax T2 FSE, DWI sequence, and Dual echo sequence were acquired. MRI parameters were as follows: 3.0 T MRI IDEAL-IQ sequence: TR/TE = 6.9 ms/3.0 ms, slice thickness of 10 mm, 200 kHZ bandwidth, FOV = 36 cm × 36 cm, matrix = 256 × 160, flip angle = 3°, NEX = 1, breath holding for less than 24 s. 1.5 T MRI IDEAL-IQ sequence: TR/TE = 13.4 ms/4.8 ms, slice thickness of 10 mm, 125 kHZ bandwidth, FOV = 36 cm × 36 cm, matrix = 256 × 160, flip angle = 5°, NEX = 1, breath holding for less than 24 s. T1WI sequence: TR/TE = 210 ms/2.4 ms. T2WI: TR/TE = 8,571 ms/100 ms. Dual-echo sequence: TR = 190 ms, TE = 2, 4.3 ms. DWI sequence: TR/TE = 7,500 ms/58 ms, NEX = 4, *b*-value = 0, 600 s/mm^2^, FOV = 42 cm × 42 cm. The images were processed using IDEAL Research software provided by the manufacturer to generate water-phase, fat-phase, in-phase, out-phase, along with R2* and fat fraction (FF) maps.

### 2.3 MRI-Acquired Fat Measurements

#### 2.3.1 Measurement of VAT and SAT

VAT and SAT were semiautomatically measured on the axial FF images by Image J (National Institutes of Health, USA), as previously described (23–25). The abdominal fat was determined at the L1–L2 level and did not include intestinal loops.

VAT was defined as intraabdominal fat (including intraperitoneal and retroperitoneal fat) bound by parietal peritoneum or transversalis fascia, excluding the vertebral column and the paraspinal muscles (23). The SAT was defined as fat superficial to the abdominal and back muscles (24). Area (cm^2^) and FF (%) of VAT and SAT were assessed. Meanwhile, visceral/subcutaneous adipose tissue area ratio (V/S) was also calculated.

In this study, the abdominal fat was measured at the L1/L2 level according to Kuk et al. (25). Most studies used the L4/L5 lumbar vertebra level for intraabdominal fat measurements to capture the highest percentage of body fat (7, 26). However, studies have shown that the cross-sectional areas of VAT and SAT measured at each level of T12-L5 are highly correlated with the volume of overall VAT and SAT (*r* = 0.89–0.98) (27). In addition, VAT significantly associated with metabolic syndrome regardless of measurement site (25, 28). Importantly, it was found that the VAT measured at L1–L2 level may predict the overall VAT more than L4/L5 level (28).

The MRI fat fraction map calculated from IDEAI-IQ was the most practical method to accurately assess ectopic fat deposition (29). In addition, several ROI sampling methods to assess ectopic fat deposition have been used in previous studies (1, 4, 24). Due to spatial heterogeneity in ectopic fat deposition, differences in the ROI sampling method can lead to fat quantification variability. To avoid such occurrences, a 3D semiautomatic segmentation method was used to assess the whole-hepatic FF, whole-pancreatic FF, and intramuscular FF in scan ranging.

#### 2.3.2 Measurement of Hepatic FF

**Figure 1:**
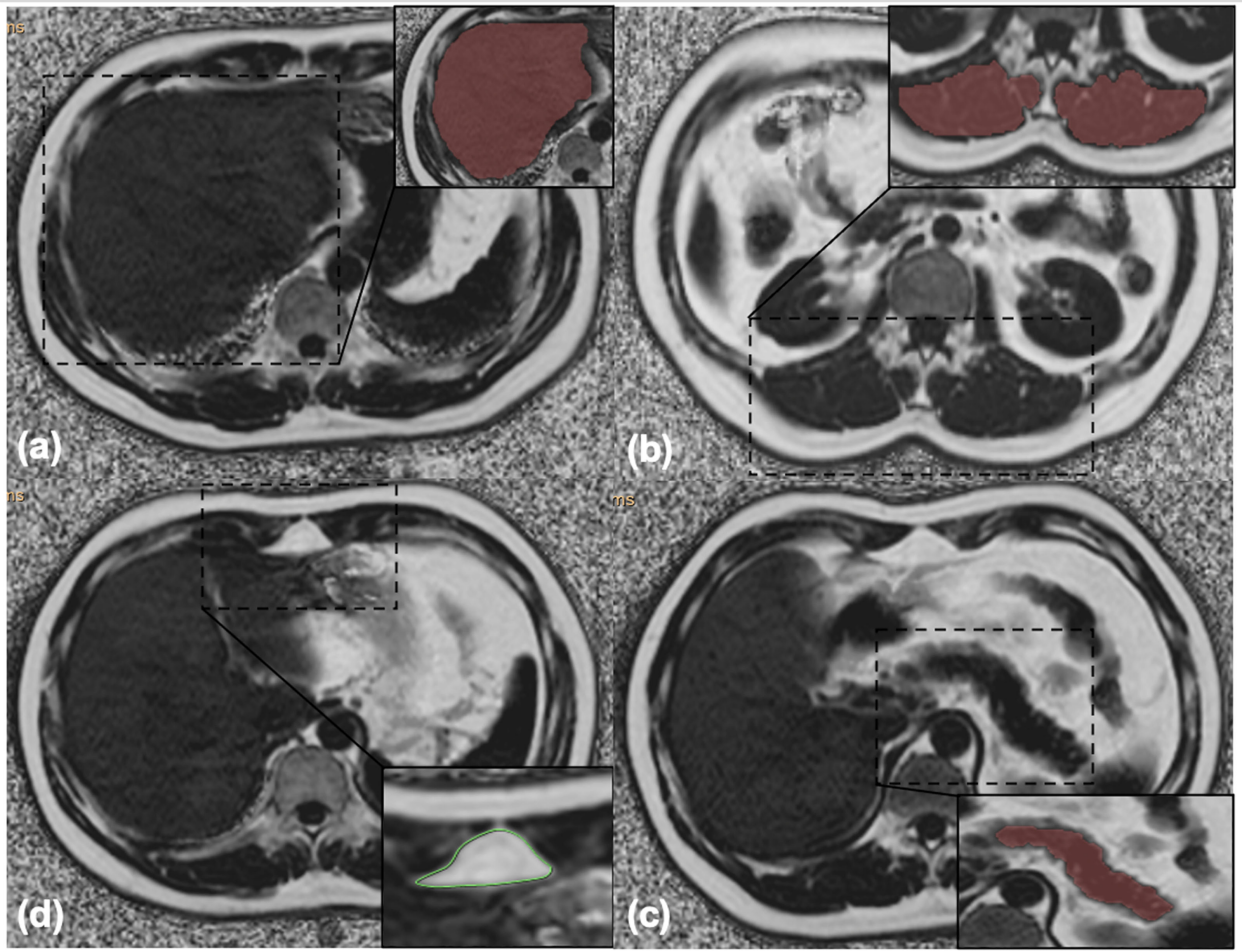
**(A)** Liver segmentation: the whole hepatic fat fraction was calculated using the 3D semiautomatic segmentation method. **(B)** Muscle segmentation: the arithmetic mean fat fraction of bilateral paraspinal muscles (including erector spinae and multifidus muscles) in the scanning range was calculated as the intramuscular fat fraction using the 3D semiautomatic segmentation method. **(C)** Pancreas segmentation: the whole pancreatic fat fraction was calculated using the 3D semiautomatic segmentation method. **(D)** Preperitoneal adipose tissue measurement: the area and the fat fraction of preperitoneal adipose tissue were measured using spline contour region of interest (ROI) method.

On the postprocessing platform (Intellispace Portal, ISP, Philips, Holland), the software algorithm defined the margins of the liver in three dimensions, and the whole liver was semiautomatically traced on FF maps. If the margins needed tweaking, the operator made corrections; if margins were included within the contours of liver segmentation, the main portal vein, inferior vena cava, and the gallbladder were manually removed. Liver was then segmented, and the whole hepatic FF was automatically calculated (**Figure 1A**).

#### 2.3.3 Measurement of Intramuscular FF

The same methodology was used to measure intramuscular FF, which referred to the arithmetic mean FF of bilateral paraspinal muscles (including erector spinae and multifidus muscles) in the scanning range (30) (**Figure 1B**).

#### 2.3.4 Measurement of Pancreatic FF

The whole pancreatic FF was calculated using the same method avoiding extrapancreatic adipose tissue and vessel (**Figure 1C**).

#### 2.3.5 Measurement of pPAT

pPAT was defined as the fat depot anteriorly seen from the anterior surface of the left lobe of the liver to the linea alba (31). pPAT area and FF were measured using the spline contour region of interest (ROI) method (**Figure 1D**).

### 2.4 Inter- and Intraobserver Variability

The intra- and interobserver variability of the MRI-acquired fat measurements was determined by repeated analysis of 30 randomly selected patients more than 4 weeks apart by the same observer and by the MRI-acquired fat measurements of the same patient by a second independent observer. Two radiologists were blinded to the grouping.

### 2.5 Cardiometabolic Risk Factors

Trained examiners measured systolic and diastolic blood pressure (BP) twice with 1–2 min intervals in the left arm after participants were seated and rested for 5 min; the mean values of the last two readings were used for analysis. Hypertension was indicated by systolic blood pressure ≥140 mmHg, diastolic blood pressure ≥90 mmHg, or on current antihypertensive drug treatment. Blood samples were collected in the morning from patients who fasted for ≥12 h prior to the blood draw. Fasting plasma glucose (FPG), TG, total cholesterol (TC), high-density lipoprotein cholesterol (HDL-C), and low-density lipoprotein cholesterol (LDL-C) were evaluated from the blood samples. FPG ≥7.0 mmol/L or current use of insulin or an oral hypoglycemic agent were classified as T2DM (32). High TG was defined as triglycerides ≥2.3 mmol/L (200 mg/dl) or on drug treatment to reduce lipid concentrations. High TC was defined as total cholesterol ≥6.2 mmol/L (240 mg/dl) or on drug treatment to reduce lipid concentrations. The cutoff point of HDL-cholesterol concentrations for defining low HDL-C was less than 1.0 mmol/L (40 mg/dl) or on drug treatment to elevate HDL-C. The cutoff point of LDL-C concentrations for defining high LDL-C was more than but equal to 4.1 mmol/L (160 mg/dl) or on drug treatment to decrease LDL-C. CCRFs were defined as the presence of two or more of these risk factors.

### 2.6 Covariates

Weight was measured in kilograms and height in meters that were then used to calculate BMI (weight divided by the square of height). Data on smoking status, alcohol intake, and family history of cardiometabolic risk factors were collected by an on-site physician-administered medical and through physical history interview during the regular clinical examination. For women, information on postmenopausal status was also collected.

### 2.7 Statistical Analysis

All data were analyzed using SPSS Ver.25.0 (SPSS Inc., Chicago, IL, USA). The Kolmogorow–Smironov test was used to test the normality of the variables in overall subjects. Normally distributed data were expressed as means ± standard deviations, and nonnormally distributed data were expressed as medians and ranges (25th and 75th percentiles). Nominal data were expressed as the frequency with percentage.

The comparisons between men and women were determined using two-sided *t*-tests or the nonparametric Mann–Whitney *U*-test for normally or nonnormally distributed data for continuous variables and the chi-square test for categorical variables.

The intraclass correlation coefficient (ICC) is defined as the ratio of the between-group variance to the total variance and is used to check the consistency or reliability of data measured multiple times on the same subject. The closer the ICC value is to 1, the closer the multiple measurement data of the same research object is, the higher the consistency of measurement results is. The generally accepted ICC evaluation criteria are as follows (33), when it is below 0.40, the consistency is poor; when it is between 0.40 and 0.59, the consistency is fair; when it is between 0.60 and 0.74, the consistency is good; and when it is between 0.75 and 1.00, the consistency is excellent.

To assess the associations between fat measurements, age and sex-adjusted correlation coefficients (*r*) among various MRI-acquired fat depots were computed. Correlation coefficients were interpreted as follows: weak, 0–0.4; moderate, 0.4–0.7; and strong, 0.7–1.0. This method was also performed to evaluate correlations of MRI-acquired fat depots and each cardiometabolic risk factor. Age-adjusted correlation analyses stratified by sex were also conducted.

Multivariable logistic and linear regression models were performed for the dichotomous and continuous outcomes, respectively. Odds ratios (ORs) from the logistic regression models and *β*-coefficients from the linear regression models, and their 95% CIs were used to assess the associations of the cardiometabolic risk factors per 1-SD increase in various MRI-acquired fat depots. Multivariable adjustments included age, sex, smoking status, current alcohol use, postmenopausal status (women only), and family history of cardiometabolic risk factors. Furthermore, an additional model-specific adjustment was applied for models as follows: antihypertensive treatment for the systolic and diastolic blood pressure models; diabetes treatment for the FPG model; lipid-lowering treatment for the TG, TC, HDL-C, and LDL-C models.

Additional models evaluated the associations between MRI-acquired fat depots and cardiometabolic risk factors after further adjustment for BMI to explore whether the associations persisted after adjusting for generalized obesity. Sex-specific multivariable-adjusted regression models were also performed.

A two-tailed *p* < 0.05 was considered to be statistically significant.

## 3 Results

### 3.1 Study Sample Characteristics

A total of 320 patients (148 men and 172 women) with a median age of 59 years (range from 19 to 90 years) and a mean BMI of 24.68 kg/m^2^ were finally included in the study. The patients’ demographic, clinical, lifestyle characteristics, and family history of cardiometabolic risk factors are shown in **Table 1**. Hypertension had the highest prevalence rate (40.94%), while high LDL-C was the least prevalent (9.38%). The prevalence rate of CCRFs was 33.75%. For fat measurements, men had higher BMI, higher SAT area, higher VAT area, higher VAT FF, higher V/S, higher pancreatic FF, higher intramuscular FF, and higher pPAT area, but lower SAT FF compared with women (all *p* < 0.05). For cardiometabolic risk factors, men had a higher prevalence of hypertension, T2DM, high TC, and low HDL-C than women (all *p* < 0.05).

**Table 1:** Clinical characteristics of the study subjects.

Characteristics	Overall patients (*n* = 320)	Men (*n* = 148)	Women (*n* = 172)	*p*-value (men vs. women)
Demographics				
Age (years)	59 (50, 65)	58 (48, 65)	60 (52, 66)	0.577
Height (m)	1.67 (1.61, 1.74)	1.74 (1.70, 1.77)	1.62 (1.60, 1.65)	<0.001
Weight (kg)	68 (60, 77)	75 (70, 82)	62.25 (58.5, 69.00)	<0.001
BMI (kg/m^2^)	24.68 ± 3.04	25.18 ± 2.80	24.25 ± 3.16	0.006
MRI-acquired fat measurements				
SAT area (cm^2^)	121.68 (94.60, 158.95)	106.19 (79.99, 126.74)	145.00 (114.65, 187.11)	<0.001
SAT FF (%)	81.73 (77.95, 84.45)	79.02 (75.62, 82.10)	83.80 (80.97, 85.67)	<0.001
VAT area (cm^2^)	141.98 (100.77, 190.36)	176.63 (128.86, 222.26)	125.22 (86.50, 162.68)	<0.001
VAT FF (%)	77.77 (74.22, 80.54)	78.37 (74.85, 80.94)	77.25 (73.69, 79.88)	0.045
V/S	1.04 (0.71, 1.62)	1.65 (1.16, 2.27)	0.76 (0.61, 1.00)	<0.001
Hepatic FF (%)	3.70 (2.70, 6.40)	3.90 (2.90, 6.60)	3.55 (2.60, 6.25)	0.415
Pancreatic FF (%)	8.10 (5.00, 12.30)	8.50 (5.60, 13.50)	7.35 (4.40, 11.65)	0.010
Intramuscular FF (%)	5.15 (3.80, 7.45)	6.00 (4.45, 8.15)	4.20 (3.30, 5.65)	<0.001
pPAT FF (%)	85.10 (80.25, 89.00)	85.20 (80.25, 88.95)	85.00 (80.15, 89.00)	0.662
pPAT area (cm^2^)	2.79 (1.97, 4.00)	3.35 (2.38, 4.71)	2.44 (1.73, 3.41)	<0.001
Cardiometabolic risk factors				
Systolic BP (mmHg)	121 (120, 140)	130 (120,140)	120 (110, 130)	0.001
Diastolic BP (mmHg)	80 (70, 80)	80 (74, 86)	80 (70, 80)	<0.001
FPG (mmol/L)	5.16 (4.73, 5.86)	5.33 (4.80, 6.29)	5.06 (4.68, 5.55)	0.014
TG (mmol/L)	1.24 (0.89, 1.88)	1.25 (0.93, 1.92)	1.22 (0.85, 1.83)	0.357
TC (mmol/L)	4.88 (4.24, 5.59)	4.62 (3.95, 5.26)	5.12 (4.44, 5.88)	<0.001
HDL-C (mmol/L)	1.27 (0.97, 1.47)	1.10 (0.91, 1.38)	1.39 (1.17, 1.61)	<0.001
LDL-C (mmol/L)	2.66 (2.20, 3.21)	2.58 (2.14, 3.10)	2.79 (2.36, 3.35)	0.008
Hypertension (*n* (%))	131 (40.94)	70 (47.29)	61 (35.47)	0.032
T2DM (*n* (%))	66 (20.63)	38 (25.68)	28 (16.28)	0.038
High TG (*n* (%))	71 (22.19)	31 (20.95)	40 (22.47)	0.620
High TC (*n* (%))	55 (17.19)	16 (10.81)	39 (21.91)	0.004
Low HDL-C (*n* (%))	104 (32.50)	63 (42.57)	41 (23.84)	<0.001
High LDL-C (*n* (%))	30 (9.38)	11 (7.43)	19 (10.67)	0.265
CCRFs (*n* (%))	108 (33.75)	61 (41.22)	47 (27.33)	0.171
Lifestyle factors				
Smoking status				<0.001
Current smoker (*n* (%))	30 (9.38)	29 (19.59)	1 (0.58)	–
Former smoker (*n* (%))	8 (2.50)	8 (5.41)	0 (0)	–
Never smoker (*n* (%))	282 (88.13)	111 (75.00)	171 (99.42)	–
Current alcohol use (*n* (%))	14 (4.38)	13 (8.78)	1 (0.58)	< 0.001
Antihypertensive treatment (*n* (%))	107 (33.44)	56 (37.84)	51 (29.65)	0.620
Diabetes treatment (*n* (%))	45 (14.16)	27 (18.24)	18 (10.47)	0.238
Lipid-lowering treatment (*n* (%))	27 (8.44)	13 (2.03)	14 (8.14)	0.836
Postmenopausal status (*n* (%))	–	–	138 (80.23)	–
Family history of cardiometabolic risk factors (*n* (%))	25 (7.81)	11 (7.43)	14 (8.14)	0.814

Data were expressed as mean ± SD, median (25th and 75th percentiles) (due to nonnormal distribution), or n (%).

BMI, body mass index; SAT, subcutaneous adipose tissue; FF, fat fraction; VAT, visceral adipose tissue; V/S, visceral/subcutaneous adipose tissue area ratio; pPAT, preperitoneal adipose tissue; BP, blood pressure; FPG, fasting plasma glucose; TG, triglycerides; TC, total cholesterol; HDL-C, high-density lipoprotein cholesterol; LDL-C, low-density lipoprotein cholesterol; T2DM, type 2 diabetes mellitus; CCRFs, clustering of cardiometabolic risk factors.

### 3.2 Consistency Analysis

The data consistency is shown in **Table S1**. The ICC values were more than 0.9, which suggested good inter-observer and intra-observer agreement.

### 3.3 Correlations Among Fat Measurements

Age- and sex-adjusted correlation coefficients among fat measurements are shown in **Table 2**. Most of the fat measurements were associated with each other (*p* < 0.05), but the strengths of these associations greatly varied. Both hepatic FF and pancreatic FF had the strongest correlations with VAT area (correlation coefficients (*r*) = 0.313 and 0.367, respectively), but intramuscular FF had the strongest correlation with SAT area (*r*-value = 0.226). Different from other MRI-acquired fat depots, VAT FF, V/S, and pPAT area were not correlated with BMI (*p* > 0.05). However, the patterns of the correlations slightly varied in both men and women **(** **Table S2****).**

**Table 2:** The age- and sex-adjusted correlations among MRI-acquired fat measurements in all patients.

MRI-acquired fat measurements	SAT area	SAT FF	VAT area	VAT FF	V/S	Hepatic FF	Pancreatic FF	Intramuscular FF	pPAT area	pPAT FF
SAT area	–	0.644^**^	0.380^**^	−0.072	−0.140^*^	0.300^**^	0.293^*^	0.226^**^	0.118^*^	0.155^*^
SAT FF	0.644^**^	–	0.313^**^	−0.128^*^	−0.123^*^	0.250^**^	0.205^*^	0.099	0.086	0.280^**^
VAT area	0.380^*^	0.313^**^	–	−0.059	0.762^**^	0.313^**^	0.367^**^	0.118^*^	0.091	0.186^*^
VAT FF	−0.072	−0.128^*^	−0.059	–	0.046	−0.041	−0.052	−0.042	0.003	0.026
V/S	−0.140^**^	−0.123^*^	0.762^**^	0.046	–	0.075	0.136^*^	−0.019	0.007	0.058
Hepatic FF	0.300^**^	0.250^**^	0.313^**^	−0.041	0.075	–	0.205^**^	0.097	0.044	0.186^**^
Pancreatic FF	0.293^**^	0.205^*^	0.367^**^	−0.052	0.136^*^	0.205^**^	–	0.169^*^	0.069	0.154^**^
Intramuscular FF	0.226^**^	0.099	0.118^*^	−0.042	−0.019	0.097	0.169^*^	–	0.043	0.048
pPAT area	0.118^*^	0.086	0.091	0.003	0.007	0.044	0.069	0.043	–	0.010
pPAT FF	0.155^*^	0.280^**^	0.186^*^	0.026	0.058	0.186^**^	0.154^**^	0.048	0.010	–

^*^p < 0.05; ^**^p < 0.001.

BMI, body mass index; SAT, subcutaneous adipose tissue; FF, fat fraction; VAT, visceral adipose tissue; V/S, visceral/subcutaneous adipose tissue area ratio; pPAT, preperitoneal adipose tissue.

### 3.4 Correlations Between the MRI-Acquired Fat Measurements and Cardiometabolic Risk Factors

The age- and sex-adjusted correlations between MRI-acquired fat measurements and continuous cardiometabolic risk factors are described in **Table 3**. For all patients, SAT area was significantly correlated with most continuous cardiometabolic risk factors (*r*-value ranging 0.124 to −0.203), except for TC and LDL-C, and had the strongest correlations with HDL-C (*r*-value = −0.203). SAT FF was only significantly correlated with HDL-C (*r*-value = −0.120). VAT area was significantly correlated with all continuous cardiometabolic risk factors (*r*-values ranging from 0.124 to 0.285) and had the strongest correlations with FPG (*r*-value = 0.285). V/S was only significantly correlated with FPG (*r*-value = 0.205). Hepatic FF was significantly correlated with most continuous cardiometabolic risk factors (*r*-value ranging from 0.140 to 0.306), except for systolic BP and HDL-C, and had the strongest correlations with TG (*r*-value = 0.306). Pancreatic FF was significantly correlated with FPG, TG, and HDL-C (*r*-value = 0.245, 0.183, and −0.193, respectively). VAT FF, intramuscular FF, and pPAT area were not significantly correlated with any continuous cardiometabolic risk factors (all *p* > 0.05). Preperitoneal FF was significantly correlated with diastolic BP, FPG, and TG (*r*-value = 0.118, 0.122, and 0.174, respectively).

**Table 3:** The age- and sex-adjusted correlations between MRI-acquired fat measurements and continuous cardiometabolic risk factors.

MRI-acquired fat measurements	Continuous cardiometabolic risk factors						
	Systolic BP	Diastolic BP	FPG	TG	TC	HDL-C	LDL-C
SAT area	0.126^*^	0.128^*^	0.124^*^	0.177^*^	0.042	−0.203^**^	0.060
SAT FF	−0.010	0.087	0.083	0.106	0.027	−0.120^*^	0.052
VAT area	0.150^*^	0.207^**^	0.285^**^	0.210^**^	0.124^*^	−0.185^*^	0.154^*^
VAT FF	0.019	0.001	−0.038	−0.027	−0.100	−0.064	−0.079
V/S	0.088	0.081	0.205^**^	0.064	0.050	−0.081	0.068
Hepatic FF	0.099	0.140^*^	0.168^*^	0.306^**^	0.148^*^	−0.104	0.156^*^
Pancreatic FF	0.081	0.097	0.245^**^	0.183^*^	0.045	−0.193^*^	0.071
Intramuscular FF	0.108	0.086	−0.021	0.030	0.020	−0.068	0.031
pPAT area	0.048	0.043	0.099	0.071	0.013	0.020	0.022
pPAT FF	0.096	0.118^*^	0.122^*^	0.174^*^	0.045	−0.101	0.032

^*^p < 0.05; ^**^p < 0.001.

BMI, body mass index; SAT, subcutaneous adipose tissue; FF, fat fraction; VAT, visceral adipose tissue; V/S, visceral/subcutaneous adipose tissue area ratio; pPAT, preperitoneal adipose tissue; BP, blood pressure; FPG, fasting plasma glucose; TG, triglycerides; TC, total cholesterol; HDL-C, high-density lipoprotein cholesterol; LDL-C, low-density lipoprotein cholesterol.

The results of correlations between the MRI-acquired fat measurements and cardiometabolic risk factors in the sex-stratified analyses are described in **Supplementary Material I**.

### 3.5 MRI-Acquired Fat Measurements as Factor Correlate With Continuous Cardiometabolic Risk Factors

**Figure 2:**
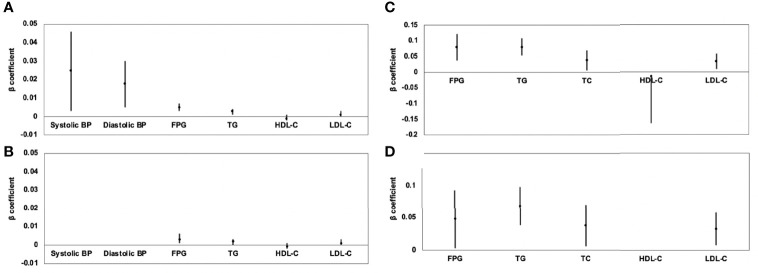
Association of VAT area **(A)** and hepatic FF **(C)** with continuous cardiometabolic risk factors and further adjustment for BMI **(B, D)** expressed by *β*-coefficients and 95% confidence intervals. For all patients, higher VAT area was associated with higher systolic BP, higher diastolic BP, higher FPG, higher TG, lower HDL-C, and higher LDL-C **(A)**; after further adjustment for BMI, these correlations continued to exist, except for systolic and diastolic BP **(B)**. Higher hepatic FF was associated with higher FPG, higher TG, higher TC, lower HDL-C, and higher LDL-C **(C)**; after further adjustment for BMI, these correlations were still significant, except for HDL-C **(D)**.

**Figure 3:**
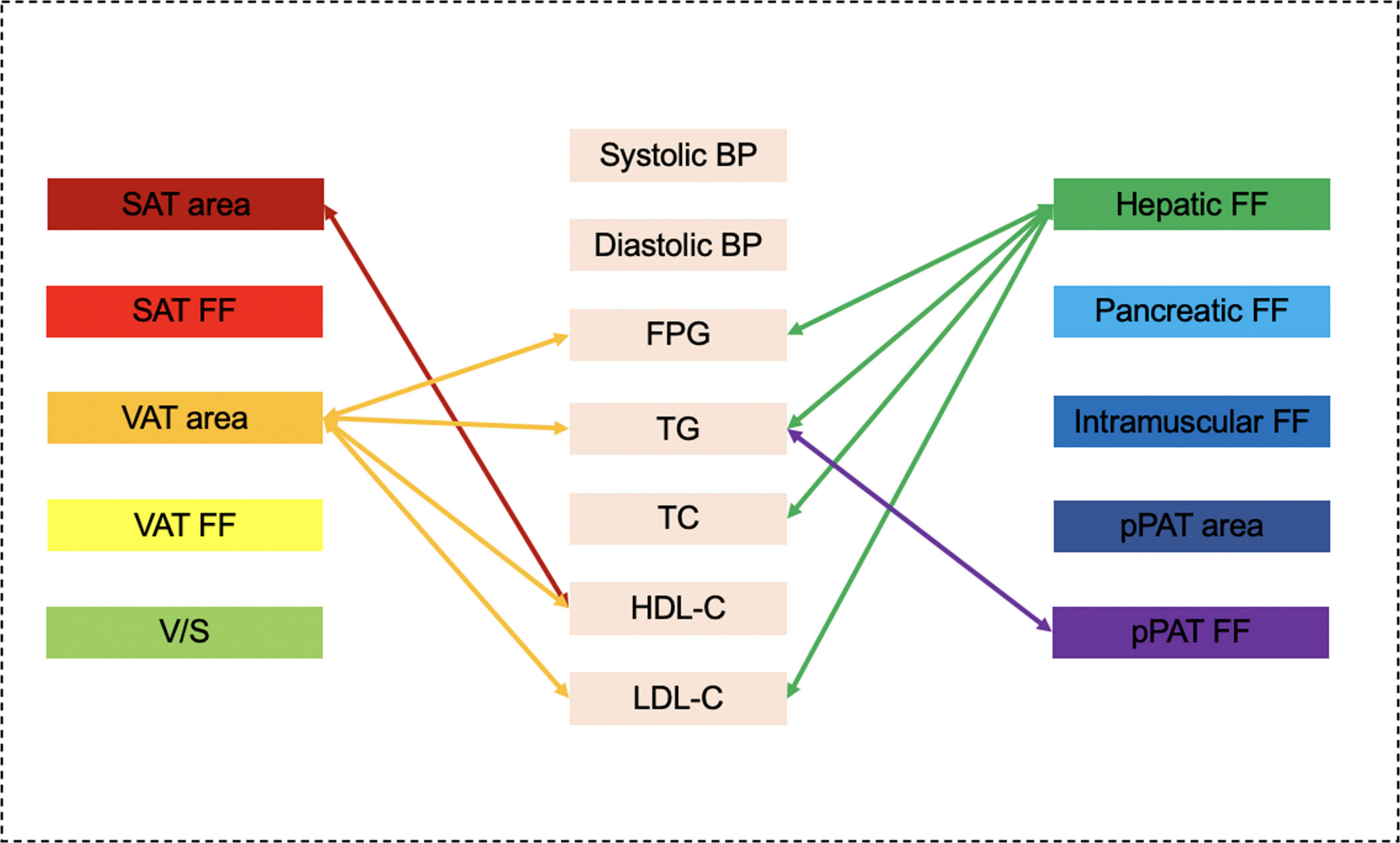
Graphical summary of the important correlates between various MRI-acquired fat measurements and continuous cardiometabolic risk factors independent of BMI shown in the present study. VAT area and hepatic fat were significantly associated with multiple continuous cardiometabolic risk factors.

Results of linear regression analyses for various MRI-acquired fat measurements for continuous cardiometabolic risk factors are shown in **Table 4**. For all patients, higher VAT area was associated with higher systolic BP, higher diastolic BP, higher FPG, higher TG, lower HDL-C, and higher LDL-C (*β* = 0.025, 0.018, 0.005, 0.003, −0.001, and 0.001, respectively). After further adjustment for BMI, these correlations continued to exist, except for systolic and diastolic BP **(** **Figures 2A, B****)**. Higher V/S was associated with higher systolic BP and higher FPG (*β* = 1.823 and 0.178, respectively), but the correlation was no longer significant after further adjustment for BMI. Higher hepatic FF was associated with higher FPG, higher TG, higher TC, lower HDL-C, and higher LDL-C (*β* = 0.080, 0.080, 0.038, −0.014, and 0.033, respectively), and after further adjustment for BMI, these correlations were still significant, except for HDL-C **(** **Figures 2C, D****)**. Although higher SAT area, higher SAT FF, higher pancreatic FF, and higher pPAT FF have a greater risk of multiple continuous cardiometabolic risk factors, after further adjustment for BMI, most of the correlations were not significant. Also, higher VAT FF, higher intramuscular FF, and higher pPAT area were not associated with any continuous cardiometabolic risk factors. The significant relationships between various MRI-acquired fat measurements and continuous cardiometabolic risk factors independent of BMI are shown in **Figure 3**.

**Table 4:** Correlations between continuous cardiometabolic risk factors and MRI-acquired fat measurements.

Risk factors	Model type^a^	Effect size	SAT area	SAT FF	VAT area	VAT FF	V/S	Hepatic FF	Pancreatic FF	Intramuscular FF	pPAT area	pPAT FF
Systolic BP^b^	MV	*β* (95% CI)	–	–	0.025 (0.003, 0.046)	–	1.823 (0.245, 3.401)	–	–	–	–	–
		*p*-value	–	–	0.023	–	0.024	–	–	–	–	–
	MV+BMI	*β* (95% CI)	–	–	–	–	–	–	–	–	–	–
		*p*-value	–	–	–	–	–	–	–	–	–	–
Diastolic BP^b^	MV	*β* (95% CI)	–	–	0.018 (0.005, 0.030)	–	–	–	–	–	–	–
		*p*-value	–	–	0.006	–	–	–	–	–	–	–
	MV+BMI	*β* (95% CI)	–	–	–	–	–	–	–	–	–	–
		*p*-value	–	–	–	–	–	–	–	–	–	–
FPG^c^	MV	*β* (95% CI)	0.004 (0.001, 0.007)	–	0.005 (0.003, 0.007)	–	0.178 (0.022, 0.333)	0.080 (0.037, 0.122)	0.033 (0.011, 0.055)	–	–	0.016 (0.000, 0.032)
		*p*-value	0.017	–	<0.001	–	0.025	<0.001	0.003	–	–	0.048
	MV+BMI	*β* (95% CI)	–	–	0.003 (0.001, 0.006)	–	–	0.048 (0.003, 0.093)	–	–	–	–
		*p*-value	–	–	0.006	–	–	0.036	–	–	–	–
TG^d^	MV	*β* (95% CI)	0.004 (0.001, 0.006)	–	0.003 (0.001, 0.004)	–	–	0.080 (0.053, 0.108)	0.020 (0.007, 0.033)	–	–	0.017 (0.007, 0.028)
		*p*-value	0.002	–	<0.001	–	–	<0.001	0.002	–	–	0.002
	MV+BMI	*β* (95% CI)	–	–	0.002 (0.000, 0.003)	–	–	0.068 (0.038, 0.098)	–	–	–	0.013 (0.002, 0.024)
		*p*-value	–	–	0.031	–	–	<0.001	–	–	–	0.016
TC^d^	MV	*β* (95% CI)	–	–	–	–	–	0.038 (0.006,0.070)	–	–	–	–
		*p*-value	–	–	–	–	–	0.020	–	–	–	–
	MV+BMI	*β* (95% CI)	–	–	–	–	–	0.038 (0.006,0.070)	–	–	–	–
		*p*-value	–	–	–	–	–	0.020	–	–	–	–
HDL-C^d^	MV	*β* (95% CI)	−0.002 (−0.003, −0.001)	−0.009 (−0.018, −0.001)	−0.001 (−0.002 −0.001)	–	–	−0.014 (−0.164, −0.018)	−0.009 (−0.014, −0.0014	–	–	–
		*p*-value	<0.001	0.037	<0.001	–	–	0.015	0.001	–	–	–
	MV+BMI	*β* (95% CI)	−0.002 (−0.003, −0.001)	–	−0.001 (−0.002 −0.001)	–	–	–	–	–	–	–
		*p*-value	<0.001	–	<0.001	–	–	–	–	–	–	–
LDL-C^d^	MV	*β* (95% CI)	–	–	0.001 (0.000, 0.003)	–	–	0.033 (0.008, 0.058)	–	–	–	–
		*p*-value	–	–	0.018	–	–	0.009	–	–	–	–
	MV+BMI	*β* (95% CI)	–	–	0.001 (0.000, 0.003)	–	–	0.033 (0.008, 0.058)	–	–	–	–
		*p*-value	–	–	0.018	–	–	0.009	–	–	–	–

Blank cells indicate that the MRI-acquired fat depots were not selected using the forward selection regression procedure.

a MV model included the following covariates: age, sex, smoking status, current alcohol use, family history of cardiometabolic risk factors, and postmenopausal status (women only). BMI was additionally adjusted for in the models labeled “MV+BMI model.”

b Antihypertensive treatment was included as a covariate for systolic and diastolic blood pressure models.

c Diabetes treatment was included as a covariate for the fasting plasma glucose model.

d Lipid-lowering treatment was included as a covariate for TG, TC, HDL-C, and LDL-C models.

BMI, body mass index; SAT, subcutaneous adipose tissue; FF, fat fraction; VAT, visceral adipose tissue; V/S, visceral/subcutaneous adipose tissue area ratio; pPAT, preperitoneal adipose tissue; BP, blood pressure; FPG, fasting plasma glucose; TG, triglycerides; TC, total cholesterol; HDL-C, high-density lipoprotein cholesterol; LDL-C, low-density lipoprotein cholesterol.

The results of MRI-acquired fat measurements as factor correlate with continuous cardiometabolic risk factors in the sex-stratified analyses are described in **Supplementary Material II**.

### 3.6 MRI-Acquired Fat Measurements as Factor Correlate With Dichotomous Cardiometabolic Risk Factors

**Figure 4:**
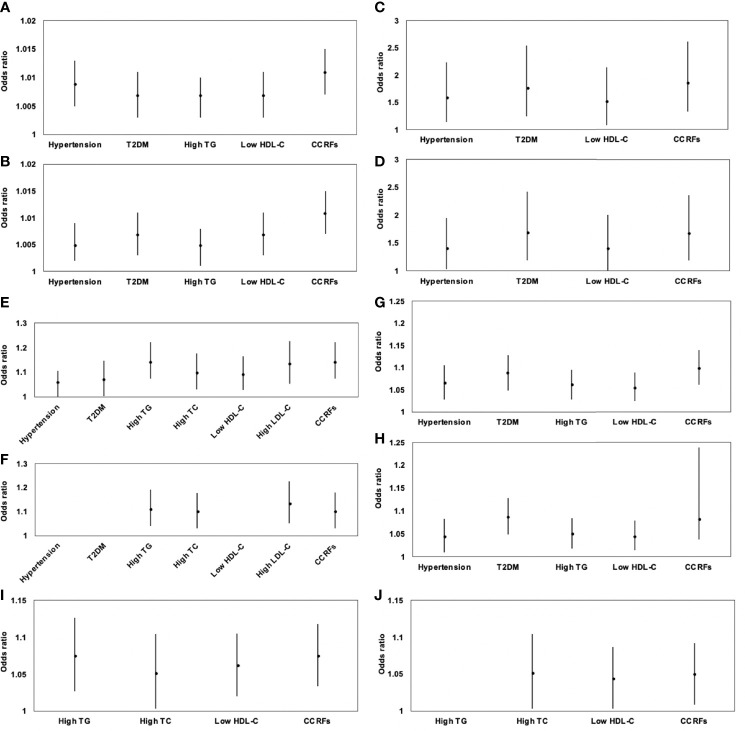
Association of VAT area **(A)**, V/S **(C)**, hepatic FF **(E)**, pancreatic FF **(G)**, and pPAT FF **(I)** with dichotomous cardiometabolic risk factors and further adjustment for BMI **(B**, **D**, **F**, **H**, **J)** expressed by odds ratios and 95% confidence intervals. VAT area was significantly associated with increased risk of hypertension, T2DM, high TG, low HDL-C, and CCRFs **(A)**; after further adjustment for BMI, these correlations continued to be significant **(B)**. V/S was significantly associated with increased risk of hypertension, T2DM, low HDL-C, and CCRFs **(C)**. After further adjustment for BMI, these correlations continued to be significant **(D)**. Hepatic FF was significantly associated with increased risk of hypertension, T2DM, high TG, high TC, low HDL-C, high LDL-C, and CCRFs **(E)**; after further adjustment for BMI, these correlations continued to be significant, except for hypertension, T2DM, and low HDL-C **(F)**. Pancreatic FF was significantly associated with increased risk of hypertension, T2DM, high TG, low HDL-C, and CCRFs **(G)**; after further adjustment for BMI, these correlations continued to be significant **(H)**. pPAT FF was significantly associated with increased risk of high TG, high TC, low HDL-C, and CCRFs **(I)**; after further adjustment for BMI, these correlations continued to be significant, except for high TG **(J)**.

**Figure 5:**
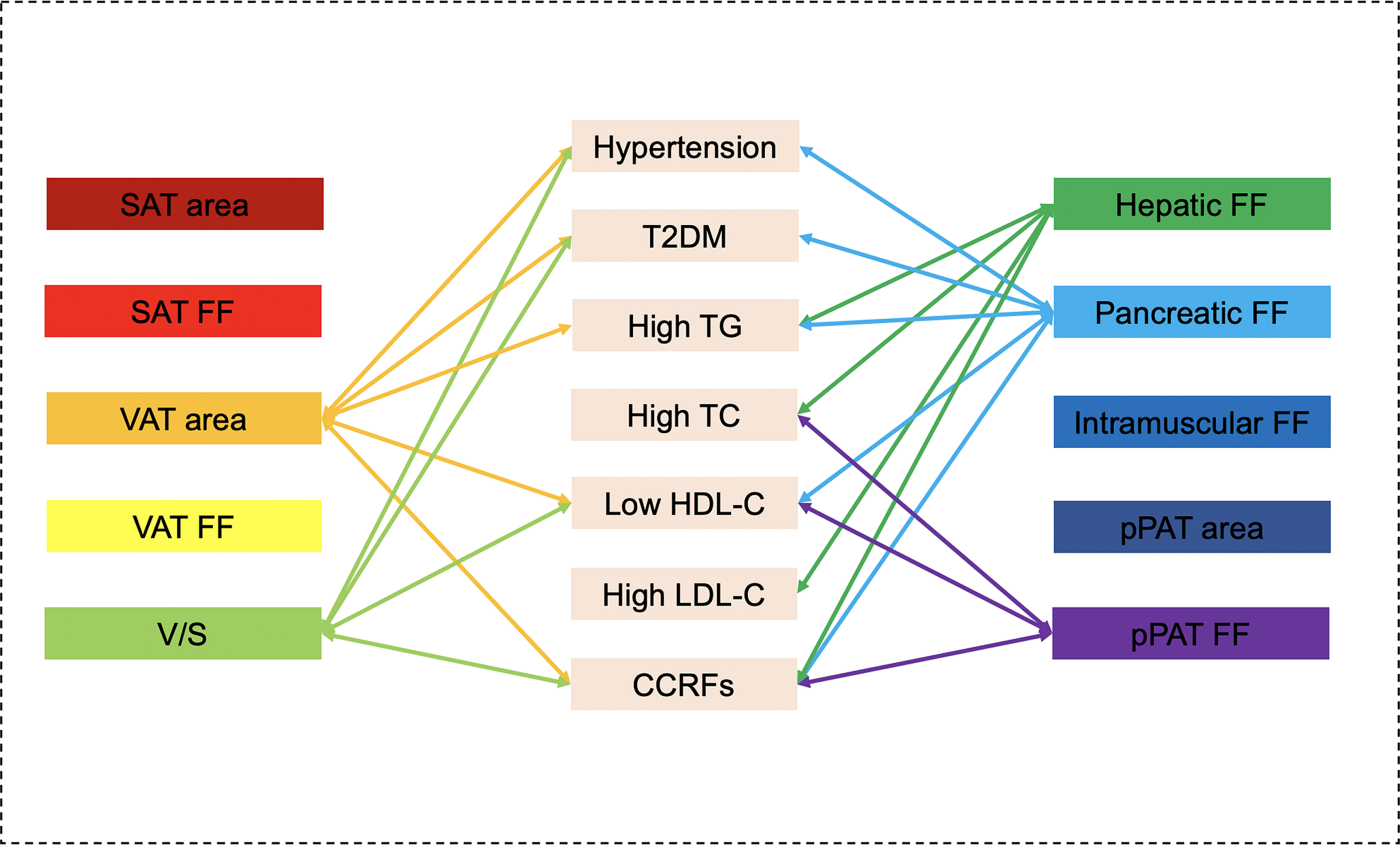
Graphical summary of the important correlates between various MR-acquired fat measurements and dichotomous cardiometabolic risk factors independent of BMI shown in the present study. VAT area, V/S, hepatic fat, pancreatic fat, and pPAT were significantly associated with multiple dichotomous cardiometabolic risk factors.

Results of sex-specific logistic regression analyses for various MRI-acquired fat measurements for dichotomous cardiometabolic risk factors are shown in **Table 5**. For all patients, VAT area was significantly associated with increased risk of hypertension, T2DM, high TG, low HDL-C, and CCRFs, with ORs of 1.009, 1.005, 1.007, 1.007, 1.007, and 1.011, respectively. After further adjustment for BMI, these correlations continued to be significant **(** **Figures 4A, B****)**. V/S was significantly associated with increased risk of hypertension, T2DM, low HDL-C, and CCRFs, with ORs of 1.597, 1.780, 1.519, and 1.865, respectively. After further adjustment for BMI, these correlations continued to be significant **(** **Figure 4C, D****)**. Hepatic FF was significantly associated with increased risk of hypertension, T2DM, high TG, high TC, low HDL-C, high LDL-C, and CCRFs, with ORs of 1.063, 1.072, 1.144, 1.102, 1.094, 1.136, and 1.144, respectively. After further adjustment for BMI, these correlations continued to be significant, except for hypertension, T2DM, and low HDL-C **(** **Figures 4E, F****)**. Pancreatic FF was significantly associated with increased risk of hypertension, T2DM, high TG, low HDL-C, and CCRFs, with ORs of 1.066, 1.088, 1.062, 1.056, and 1.100, respectively. After further adjustment for BMI, these correlations continued to be significant **(** **Figures 4G, H****)**. pPAT FF was significantly associated with increased risk of high TG, high TC, low HDL-C, and CCRFs, with ORs of 1.075, 1.052, 1.062, and 1.075, respectively. After further adjustment for BMI, these correlations continued to be significant, except for high TG **(** **Figures 4I, J****)**. Although SAT area and SAT FF were significantly associated with increased risk of multiple dichotomous cardiometabolic risk factors, after further adjustment for BMI, most of the correlations were not significant. In addition, VAT FF, intramuscular FF, and pPAT area were not significantly associated with any dichotomous cardiometabolic risk factors. The significant relationships between various MRI-acquired fat measurements and dichotomous cardiometabolic risk factors independent of BMI were shown in **Figure 5**.

**Table 5:** Correlations between dichotomous cardiometabolic risk factors and MRI-acquired fat measurements.

Risk factors	Model type^a^	Effect size	SAT area	SAT FF	VAT area	VAT FF	V/S	Hepatic FF	Pancreatic FF	Intramuscular FF	pPAT area	pPAT FF
Hypertension	MV	OR (95% CI)	1.009 (1.004, 1.015)	–	1.009 (1.005, 1.013)	0.999 (0.997, 1.001)	1.597 (1.142, 2.232)	1.063 (1.000, 1.130)	1.066 (1.028, 1.106)	–	1.041 (0.992, 1.092)	–
		*p*-value	0.001	–	<0.001	0.430	0.006	0.049	0.001	–	0.103	–
	MV+BMI	OR (95% CI)	–	–	1.005 (1.002, 1.009)	–	1.413 (1.027 1.945)	–	1.046 (1.009, 1.083)	–	–	–
		*p*-value	–	–	0.006	–	0.034	–	0.013	–	–	–
T2DM	MV	OR (95% CI)	–	–	1.007 (1.003, 1.011)	–	1.780 (1.247, 2.541)	1.072 (1.003, 1.147)	1.088 (1.049, 1.128)	–	–	–
		*p*-value	–	–	<0.001	–	0.001	0.041	<0.001	–	–	–
	MV+BMI	OR (95% CI)	–	–	1.007 (1.003, 1.011)	–	1.693 (1.185, 2.420	–	1.088 (1.049, 1.128)	–	–	–
		*p*-value	–	–	<0.001	–	0.004	–	<0.001	–	–	–
High TG	MV	OR (95% CI)	1.006 (1.001, 1.011)	–	1.007 (1.003, 1.010)	–	–	1.144 (1.073, 1.221)	1.062 (1.029, 1.095)	–	–	1.075 (1.027, 1.126)
		*p*-value	0.025	–	0.001	–	–	<0.001	<0.001	–	–	0.002
	MV+BMI	OR (95% CI)	–	–	1.005 (1.001, 1.008)	–	–	1.113 (1.040, 1.191)	1.051 (1.018, 1.084)	–	–	–
		*p*-value	–	–	0.017	–	–	0.002	0.002	–	–	–
High TC	MV	OR (95% CI)	–	–	–	–	–	1.102 (1.031, 1.177)	–	–	–	1.052 (1.003, 1.104)
		*p*-value	–	–	–	–	–	0.004	–	–	–	0.036
	MV+BMI	OR (95% CI)	–	–	–	–	–	1.102 (1.031, 1.177)	–	–	–	1.052 (1.003, 1.104)
		*p*-value	–	–	–	–	–	0.004	–	–	–	0.036
Low HDL-C	MV	OR (95% CI)	1.009 (1.003, 1.014)	1.073 (1.016, 1.133)	1.007 (1.003, 1.011)	–	1.519 (1.076, 2.144)	1.094 (1.027, 1.165)	1.056 (1.025, 1.089)	–	–	1.062 (1.020, 1.105)
		*p*-value	0.002	0.012	<0.001	–	0.018	0.005	<0.001	–	–	0.003
	MV+BMI	OR (95% CI)	1.009 (1.003, 1.014)	–	1.007 (1.003, 1.011)	–	1.420 (1.006, 2.004)	–	1.046 (1.015, 1.079)	–	–	1.044 (1.003, 1.087)
		*p*-value	0.002	–	<0.001	–	0.046	–	0.004	–	–	0.036
High LDL-C	MV	OR (95% CI)	–	–	–	–	–	1.136 (1.052, 1.227)	–	–	–	–
		*p*-value	–	–	–	–	–	0.001	–	–	–	–
	MV+BMI	OR (95% CI)	–	–	–	–	–	1.136 (1.052, 1.227)	–	–	–	–
		*p*-value	–	–	–	–	–	0.001	–	–	–	–
CCRFs	MV	OR (95% CI)	1.008 (1.003, 1.013)	–	1.011 (1.007, 1.015)	–	1.865 (1.330, 2.614)	1.144 (1.073, 1.221)	1.100 (1.061, 1.140)	–	–	1.075 (1.034, 1.118)
		*p*-value	0.003	–	<0.001	–	<0.001	<0.001	<0.001	–	–	<0.001
	MV+BMI	OR (95% CI)	–	–	1.011 (1.007, 1.015)	–	1.675 (1.191, 2.356)	1.102 (1.030, 1.179)	1.084 (1.038, 1.238)	–	–	1.050 (1.009, 1.092)
		*p*-value	–	–	<0.001	–	0.003	0.005	0.005	–	–	0.016

Blank cells indicate that the MRI-acquired fat depots were not selected via the forward LR selection regression procedure.

a MV model included the following covariates: age, sex, smoking status, current alcohol use, family history of cardiometabolic risk factors, and postmenopausal status (women only). BMI was additionally adjusted for in the models labeled “MV+BMI model.”

BMI, body mass index; SAT, subcutaneous adipose tissue; FF, fat fraction; VAT, visceral adipose tissue; V/S, visceral/subcutaneous adipose tissue area ratio; pPAT, preperitoneal adipose tissue; BP, blood pressure; FPG, fasting plasma glucose; TG, triglycerides; TC, total cholesterol; HDL-C, high-density lipoprotein cholesterol; LDL-C, low-density lipoprotein cholesterol; T2DM, type 2 diabetes mellitus; CCRFs, clustering of cardiometabolic risk factors.

The results of MRI-acquired fat measurements as factor correlate with dichotomous cardiometabolic risk factors in the sex-stratified analyses are described in **Supplementary Material II**.

## 4 Discussion

In this study, the main observations can be summarized as follows: firstly, most fat measurements were associated with each other, but the strength of these associations greatly varied. Both hepatic FF and pancreatic FF had the strongest correlations with the VAT area, but intramuscular FF had the strongest correlation with SAT area. Secondly, we found VAT area, V/S, hepatic fat, and pancreatic fat rather than SAT area were significantly associated with multiple cardiometabolic risk factors. However, the patterns of these correlations varied by sex and specific risk factors. In addition, VAT and SAT FF were only significantly associated with multiple cardiometabolic risk factors in women. Finally, we also identified differential correlations between pPAT and cardiometabolic risk factors, especially in men.

### 4.1 SAT, VAT, and Cardiometabolic Risk Factors

It is well documented that SAT and VAT are associated with different cardiometabolic risk factors; in particular, a more adverse effect may be attributed to VAT (8, 23, 32). Similarly, in this study, we found that VAT area and V/S were significantly associated with multiple cardiometabolic risk factors and these correlations remained after adjustment for BMI. SAT area was significantly associated with multiple cardiometabolic risk factors, but these correlations were no longer statistically significant after adjustment for BMI. Thus, the VAT area has a pathological role in developing cardiometabolic risk factors, whereas SAT area does not contribute to cardiometabolic risk factors beyond a measure of generalized adiposity. This could be due to several reasons: first, VAT has metabolic properties that are distinct from SAT (34); second, VAT is constantly releasing free fatty acids into portal circulation. Excessive intake of free fatty acids by hepatocytes can lead to insulin resistance and systemic hyperinsulinemia (35); third, excess VAT can lead to leptin resistance and increased leptin secretion (36); and fourth, VAT is a marker of ectopic fat deposition (24, 37). Our result suggests that VAT may be a better indicator for the risk of cardiometabolic risk factors. Thus, reduced VAT deposition risk may have beneficial influences in cardiometabolic risk factor control.

Interestingly, our results revealed that SAT FF and VAT FF were associated with multiple cardiometabolic risk factors independent of BMI in women. Adipose tissue with higher lipid content and lipolytic activity can increase systemic free fatty acids (38) and can also induce whole-body insulin resistance (39) and endothelial dysfunction (40). Thus, an increased fat fraction of adipose tissue may reflect worsening fat quality, which may concur with adipocyte hyperplasia and hypertrophy (41), eventually leading to an increased risk of cardiometabolic risk factors. To the best of our knowledge, this is the first study that assessed fat quality in VAT and SAT FF *via* MRI fat fraction maps to explore the association between VAT and SAT FF and cardiometabolic risk factors. Nevertheless, this finding was only observed in women in the present study. It may be explained by sexual dimorphism in the process of adipose tissue remodeling (41) and cardioprotective effects of estrogen before and after menopause (42). The precise mechanism for these sex differences is not clear and remains to be elucidated in future studies.

### 4.2 Hepatic Fat and Cardiometabolic Risk Factors

Several studies have reported that hepatic fat was associated with hypertension, T2DM, and dyslipidemia (32, 43). This may be explained by the joint role of VAT and hepatic fat in glucose and lipid metabolism (44). Because the portal vein drains most VAT, the hyperlipolytic state of adipocytes associated with VAT exposes the liver to high concentrations of free fatty acids and glycerol, leading to several impairments in liver metabolism, such as increased production of triglyceride-rich lipoproteins, as well as increased production of hepatic glucose (45). In the presence of hepatic steatosis, various intermediate lipid moieties generated during triglyceride synthesis (e.g., diacylglycerols and ceramide) have been shown to promote lipotoxicity and enhance hepatic insulin resistance (46, 47), likely by inhibiting insulin signaling pathways (48, 49). In addition, hepatic steatosis can accelerate the lipolysis and the secretion of very low-density lipoprotein, leading to dyslipidemia (50). Decreased insulin sensitivity and increased glucose production lead to decreased islet cell function and hyperglycemia (51), as well as increased release of inflammatory factors (such as IL-6, TNF-α, and C-reactive protein) (52, 53). Similarly, our study showed that hepatic FF was mainly associated with multiple cardiometabolic risk factors related to dyslipidemia traits, including high TG, high TC, high LDL-C, as well as measures of FPG, TG, TC, and LDL-C independent of BMI. This finding supports that hepatic fat may be associated with glycolipid regulation and metabolism. Thus, hepatic fat may be useful to identify those with higher risk of cardiometabolic risk factors.

### 4.3 Pancreatic Fat and Cardiometabolic Risk Factors

It was found that pancreatic fat bears adverse effects on the cardiometabolic health. In agreement with findings from previous studies, we found that pancreatic FF was significantly associated with multiple cardiometabolic risk factors, including hypertension, T2DM, high TG, low HDL-C, and CCRFs, and the correlations remained after adjustment for BMI. Zhou et al. (54) reported similarly a significant correlation of pancreatic steatosis with abdominal obesity, hypertension, hyperglycemia, and hypertriglyceridemia in a Chinese population. Bi et al. (55) systematically reviewed the association between pancreatic steatosis and metabolic comorbidities and indicated that pancreatic steatosis was significantly associated with an increased risk of metabolic syndrome and its components. However, the results in the literature showing correlations between pancreatic FF and cardiometabolic risk factors are often inconsistent, as previous studies found no significant differences in pancreatic fat fraction among subjects with normal glucose tolerance, prediabetes, and T2DM (56). These inconsistencies may be related to the small sample size, heterogeneous distribution of pancreatic fat, and genetic factors. Different from previous studies, pancreatic fat was more accurately evaluated using the 3D semiautomatic segmentation method in MRI fat fraction map in our study because of avoiding the effects of the heterogeneous distribution of pancreatic fat. Our results further confirm that assessment and monitoring of pancreatic fat may be used in the prediction of cardiometabolic risk factors and their early prevention.

### 4.4 Intramuscular Fat and Cardiometabolic Risk Factors

As with other ectopic fat depots, ectopic muscle fat has the potential of impairing insulin action through the inhibition of insulin signaling by lipotoxic diacylglycerols and ceramide and cause insulin resistance (57). Also, secretions of skeletal muscle adipocytes are able to impair insulin action and signaling of muscle fibers (58). In addition, T cells and macrophages accumulate in skeletal muscle fat of mice with diet-induced obesity. T cells and macrophages further impair metabolic functions of skeletal muscle cells through a paracrine mechanism (59). Yet, we found that it was not significantly correlated with any cardiometabolic risk factors (except for diastolic BP in women) in our study, which may be because diet and exercise can individually affect intramuscular FF (60). Further studies are required to address the role of intramuscular FF on cardiometabolic risk factors.

### 4.5 pPAT and Cardiometabolic Risk Factors

pPAT is a less-explored abdominal depot, and its nature has been debated. Although pPAT has sometimes been defined as VAT, it is actually SAT that is not located intraperitoneally and is connected to the systemic circulation rather than the portal circulation. Compared with VAT and SAT, adipose-derived stem/stromal cells derived from pPAT revealed highest capacity to generate new adipocytes by adipogenesis and low proinflammatory profile (61). Nevertheless, pPAT behaves like VAT and is correlated positively with hypertension, dyslipidemia, insulin resistance, cardiovascular disease risks, and obesity (5, 31). Compared with previous research, we found that pPAT area was significantly associated with hypertension, T2DM, high TG, low-HDL-C, CCRFs, and measures of TG and HDL-C independent of BMI in men rather than women. pPAT FF was significantly associated with low HDL-C and a measure of TG in women and CCRFs in men. Yet, the precise mechanism for this sex difference for pPAT is not clear. pPAT may play an important role in the occurrence and development of cardiovascular metabolic risk factors and the underlying mechanism remains to be elucidated in the future studies.

Our results showed that there was a BMI-independent association between ectopic fat depots and cardiometabolic risk factors, suggesting that ectopic fat depots might be more adequate indicators for the evaluation of cardiometabolic risk factors because BMI can neither reflect the regional body fat distribution nor distinguish between muscle and adipose tissue. Importantly, obesity is heterogeneity. Between 10% and 30% of obese individuals (defined by BMI) have been characterized as metabolically healthy obese. Yet, some nonobese individuals show evidence of metabolic complications typical for obesity (62). Thus, the use of BMI is only an approximation of the evaluation of the amount of fat mass and is inadequate to reflect the association between the obesity and cardiometabolic risk factors. However, the results about the strength of associations of fat depots and BMI with cardiometabolic risk factors are still inconsistent. Previous studies found that associations between fat depots and cardiometabolic risk factors were not independent of BMI and BMI seemed stronger associated with some indicators of cardiometabolic risk factors (63–65). Several potential factors may have contributed to this inconsistency. First, sex‐related differences: some studies showed that BMI may adequately capture cardiometabolic risk in men but not in women (63, 64). The exact mechanism underlying the sex difference is not known but may be related to the greater effect of free fatty acid mobilization from VAT into the hepatic portal circulation in women than in men (66). Second, racial/ethnic disparities: for each 1-unit increase in BMI, Asians had higher risk of hypertension and T2DM compared with non-Hispanic whites, Hispanics, and non-Hispanic blacks, indicating incremental weight gain in Asians is more detrimental (67). In addition, there are also racial/ethnic differences in prevalence of obesity, VAT, and NAFLD (68, 69). Due to genetic differences, NAFLD occurs not only in people with high BMI but also in people with low BMI. It was found that the G allele of PNPLA3 rs738409 was associated with increased risk of NAFLD in Asians (70) as well as in nonobese or lean individuals (71). Previous study showed that PNPLA3 polymorphism was associated with the rate of T2DM in Japanese population (72).

### 4.6 Strengths and Limitations

This study has some strengths. Initially, MRI fat fraction map was used to accurately evaluate the ectopic fat deposition in different parts of the abdomen, including VAT, SAT, pPAT, whole hepatic fat, whole pancreatic fat, and bilateral paraspinous muscle fat. Furthermore, it was the first paper to evaluate the VAT FF and SAT FF by MRI fat fraction map to explore the associations between VAT FF, SAT FF and cardiovascular metabolic risk factors. Finally, the subgroup analysis of gender stratification was performed to explore the gender differences in the association between various ectopic fat deposition and cardiovascular metabolic risk factors.

This study has several limitations. First, this was a cross-sectional study; thus, the causal relationship of MRI-acquired fat depots with cardiometabolic risk factors cannot be inferred. Well-designed prospective cohort studies are needed to elaborate the causality in the future. Second, selection bias was a major limitation of this study. Third, the sample size was small because of high cost of MRI measurements as well as strict exclusion criteria. Fourth, the intramuscular FFs in most previous studies were measured at L3 level (1, 3); however, we measured it using 3D semiautomatic segmentation ranging from T4 to T12. Fifth, data on mortality and exercise factors were unavailable in the current study; thus, we cannot evaluate these factors” influences on the observed associations. Sixth, compared with Asians, other ethnicities could have different types of ectopic fat deposition; therefore, the associations with cardiometabolic risk factors may be different, which limits the generalization of the presented findings beyond Asian populations. Sixth, FPG ≥7.0 mmol/L was used as a single diagnostic criterion for T2DM (32), which may have an impact on the results. Finally, those should be considered preliminary results. We will continue to conduct combined analysis of significant various MRI-acquired fat depots according to sex and specific risk factors, and then obtain personalized models in prediction, early prevention or therapeutic intervention of cardiometabolic risk factors in the further study.

### 4.7 Clinical Implications

This study adds an important facet to the obesity study spectrum in which, within the heterogeneity of abdominal fat distribution, VAT, hepatic fat, pancreatic fat, and pPAT were associated with cardiovascular metabolic risk factors independently of BMI. Regarding the preventive significance of this study, our findings support that only making recommendations based on BMI may lead to misclassification of high-risk individuals as “metabolically healthy obesity.” Those who are “metabolically unhealthy nonobese” and “metabolically unhealthy normal weight” will be misclassified as people with lower risk. Our study also demonstrated the patterns of these correlations varied by gender, supporting that gender should be considered an important point in classifying individuals at the high risk of cardiometabolic abnormalities. In addition, the result showed that pPAT should not be overlooked as pathologic fat depots contributing to cardiometabolic risk factors. It is necessary to explore the biology of various ectopic fat storage to precisely decipher the pathogenicity of excess adiposity in future studies.

## 5 Conclusion

VAT area, V/S, hepatic fat, pancreatic fat, and pPAT rather than SAT area were significantly associated with multiple cardiometabolic risk factors. However, the patterns of these correlations varied by sex and specific risk factors. In addition, VAT and SAT FF were only significantly associated with multiple cardiometabolic risk factors in women. These findings broaden the understanding of the association between ectopic fat deposition and cardiometabolic risk factors, thus further clarifying the heterogeneity of obesity.

## Data Availability Statement

The original contributions presented in the study are included in the article/**Supplementary Material**. Further inquiries can be directed to the corresponding author.

## Ethics Statement

The studies involving human participants were reviewed and approved by the First Affiliated Hospital of Dalian Medical University. Written informed consent for participation was not required for this study in accordance with the national legislation and the institutional requirements. Written informed consent was not obtained from the individual(s) for the publication of any potentially identifiable images or data included in this article.

## Author Contributions

Data analysis and interpretation, study design, manuscript writing, and manuscript approval were performed by Q-HZ, L-HX, L-HC, YZ, H-NZ, and A-LL, and they are accountable for all aspects of the work. MRI data and clinical data analysis and interpretation, statistical analysis, and manuscript approval were performed by J-HL, A-LC, YJ, and NW. MRI data analysis and manuscript approval were performed by Q-WS and A-LL. Statistical analysis and manuscript approval were performed by L-ZX. All authors read and approved the final manuscript.

## Conflict of Interest

Author L-ZX was employed by GE Healthcare, Beijing, China.

The remaining authors declare that the research was conducted in the absence of any commercial or financial relationships that could be construed as a potential conflict of interest.

## Publisher’s Note

All claims expressed in this article are solely those of the authors and do not necessarily represent those of their affiliated organizations, or those of the publisher, the editors and the reviewers. Any product that may be evaluated in this article, or claim that may be made by its manufacturer, is not guaranteed or endorsed by the publisher.

## Abbreviations

BIA, bioelectrical impedance analysis; BMI, body mass index; BP, blood pressure; CCRFs, clustering of cardiometabolic risk factors; CT, computed tomography; DXA, dual-energy x-ray absorptiometry; FF, fat fraction; FPG, fasting plasma glucose; HDL-C, high-density lipoprotein cholesterol; ICC, intraclass correlation coefficient; IDEAL-IQ, iterative decomposition of water and fat with echo asymmetry and least-squares estimation-iron quantification; LDL-C, low-density lipoprotein cholesterol; MRI, magnetic resonance imaging; ORs, odds ratios; pPAT, preperitoneal adipose tissue; ROI, region of interest; SAT, subcutaneous adipose tissue; TC, total cholesterol; TG, triglycerides; T2DM, type 2 diabetes mellitus; VAT, visceral adipose tissue; WC, waist circumference; WHR, waist-to-hip ratio; V/S, visceral/subcutaneous adipose tissue area ratio.
