# Supplementary material for: Magnetic Resonance Imaging Assessment of Abdominal Ectopic Fat Deposition in Correlation With Cardiometabolic Risk Factors
Source: Front Endocrinol (Lausanne). 2022 Mar 30;13:820023. doi: 10.3389/fendo.2022.820023 (PMC9005896; doi:10.3389/fendo.2022.820023)
Supplement: Supplementary file 1 [file DataSheet_1.docx]

**Supplemental Materials**

**I Correlations between the MRI-acquired fat measurements and cardiometabolic risk factors**

In the sex-stratified analyses, SAT area was significantly correlated with systolic and diastolic BP (r-value = 0.190 and 0.349, respectively) in men. SAT FF was only significantly correlated with diastolic BP (r-value = 0.206). VAT area was significantly correlated with diastolic BP, FPG, and HDL-C (r-value = 0.214, 0.343, and -0.247, respectively). VAT FF and intramuscular FF were not significantly correlated with any continuous cardiometabolic risk factors (all P > 0.05). V/S was only significantly correlated with FPG (r-value = 0.265). Hepatic FF was significantly correlated with FPG and TG (r-value = 0.167 and 0.240, respectively). Pancreatic FF was significantly correlated with FPG, TG, and HDL-C (r-value = 0.258, 0.167, and -0.233, respectively). pPAT area was significantly correlated with most continuous cardiometabolic risk factors (r-value ranging from 0.190 to 0.308), except for TC and LDL-C, and had the strongest correlations with FPG (r-value = 308). pPAT FF was significantly correlated with diastolic BP (r-value = 0.174).

In women, SAT area was significantly correlated with TG and HDL-C (r-value = 0.263 and -0.237, respectively). SAT FF was also significantly correlated with TG and HDL-C (r-value = 0.152 and -0.181, respectively). VAT area was significantly correlated with most continuous cardiometabolic risk factors (r-value ranging from 0.197 to 0.337), except for HDL-C, and had the strongest correlations with TG (r-value = 0.337). VAT FF was significantly correlated with most continuous cardiometabolic risk factors (r values ranging from 0.191 to 0.454), except for diastolic BP, and had the strongest correlations with TG (r-value = 0.454). V/S was significantly correlated with most continuous cardiometabolic risk factors (r-value ranging from 0.173 to 0.216), except for HDL-C, and had the strongest correlations with LDL-C (r-value = 0.216). Hepatic FF was significantly correlated with FPG, TG, TC and LDL-C (r-value = 0.175, 0.378, 0.184 and 0.191, respectively). Pancreatic FF was significantly correlated with FPG, TG, and HDL-C (r-value = 0.235, 0.205, and -0.169, respectively). Intramuscular FF was only significantly correlated with diastolic BP (r-value = 0.188). pPAT area was not significantly correlated with any continuous cardiometabolic risk factors (all P > 0.05). pPAT FF was only significantly correlated with TG (r-value = 0.250) **(Table S3).**

**II MRI-acquired fat measurements as factor correlate with continuous cardiometabolic risk factors**

In the sex-stratified analyses, higher pPAT area was associated with higher diastolic BP, higher FPG, higher TG, and lower HDL-C (β = 1.045, 0.275, 0.121, and -0.035, respectively); after further adjustment for BMI, all of those correlations continued to be significant except for diastolic BP and FPG in men **(Figure S1)**. Higher SAT area, higher SAT FF, higher VAT, higher V/S, and higher pancreatic FF have a greater risk of several continuous cardiometabolic risk factors, after further adjustment for BMI, most of the correlations were not significant. In addition, higher VAT FF, higher intramuscular FF, and pPAT FF were not associated with any continuous cardiometabolic risk factors.

In women, higher VAT area was associated with higher FPG, higher TG, higher TC, lower HDL-C, and higher LDL-C (β = 0.008, 0.007, 0.004, -0.002, and 0.005, respectively). After further adjustment for BMI, those correlations continued to be significant **(Figure S2a-S2b)**. Higher VAT FF was associated with higher FPG, higher TG, higher TC, lower HDL-C, and higher LDL-C (β = 0.063, 0.081, 0.051, -0.027, and 0.058, respectively). After further adjustment for BMI, those correlations continued to be significant, except for higher FPG **(Figure S2c-S2d)**. Higher V/S was associated with higher systolic BP, higher diastolic BP, higher TG, higher TC, and higher LDL-C (β = 12.167, 7.713, 0.984, 1.045, and 1.126, respectively). After further adjustment for BMI, those correlations continued to be significant, except for higher systolic BP **(Figure S2e-S2f)**. Higher hepatic FF was associated with higher FPG, higher TG, higher TC, and higher LDL-C (β = 0.087, 0.083, 0.059, 0.049, respectively). After further adjustment for BMI, those correlations continued to be significant **(Figure S2g-S2h)**. After further adjustment for BMI higher SAT area, higher SAT FF, higher pancreatic FF, intramuscular FF, and higher pPAT FF was associated with few continuous cardiometabolic risk factors. In addition, the pPAT area was not associated with any continuous cardiometabolic risk factors (**Table S4**). The significant relationships between various MRI-acquired fat measurements and continuous cardiometabolic risk factors independent of BMI in men and women were shown in **Figure S3.**

**III MRI-acquired fat measurements as factor correlate with dichotomous cardiometabolic risk factors**

In the sex-stratified analyses, in men, VAT area was significantly associated with increased risk of hypertension, T2DM, high TG, low HDL-C, and CCRFs, with ORs of 1.009, 1.007, 1.006, 1.011, and 1.011, respectively. After further adjustment for BMI, those correlations continued to be significant **(Figure S4a-S4b)**. V/S was significantly associated with increased risk of hypertension, T2DM, low HDL-C, and CCRFs, with ORs of 1.451, 1.888, 2.034, and 2.437, respectively. After further adjustment for BMI, those correlations continued to be significant **(Figure S4c-S4d)**. Hepatic FF was significantly associated with increased risk of high TG and low HDL-C, with ORs of 1.126 and 1.144, respectively. After further adjustment for BMI, those correlations continued to be significant. Pancreatic FF was significantly associated with increased risk of T2DM, high TG, low HDL-C, and CCRFs, with ORs of 1.102, 1.075, 1.060, and 1.090, respectively. After further adjustment for BMI, those correlations continued to be significant **(Figure S4e-S4f)**. pPAT area was significantly associated with increased risk of hypertension, T2DM, high TG, low HDL-C, and CCRFs, with ORs of 1.496, 1.262, 1.254, 1.404, and 1.288, respectively. After further adjustment for BMI, those correlations continued to be significant **(Figure S4g-S4h)**. pPAT FF was only significantly associated with increased risk of CCRFs, with an OR of 1.067. After further adjustment for BMI, the correlation continued to be significant. SAT area was significantly associated with increased risk of hypertension, with an OR of 1.011. After further adjustment for BMI, the correlation was no longer significant. In addition, SAT FF, VAT FF, and intramuscular FF were not significantly associated with an increased risk of any dichotomous cardiometabolic risk factors.

In women, VAT area was significantly associated with increased risk of hypertension, T2DM, high TG, high TC, high LDL-C, and CCRFs, with ORs of 1.013, 1.012, 1.016, 1.013, 1.013, and 1.017, respectively. After further adjustment for BMI, those correlations continued to be significant **(Figure S5a-S5b)**. VAT FF was significantly associated with increased risk of hypertension, high TG, high TC, low HDL-C, high LDL-C, and CCRFs, with ORs of 1.175, 1.250, 1.181, 1.150, 1.134, and 1.221, respectively. After further adjustment for BMI, those correlations continued to be significant **(Figure S5c-S5d)**. V/S was significantly associated with increased risk of hypertension, T2DM, high TG, high TC, high LDL-C, and CCRFs, with ORs of 5.470, 5.159, 8.559, 19.919, 26.457, and 9.404, respectively. After further adjustment for BMI, those correlations continued to be significant **(Figure S5e-S5f)**. Hepatic FF was significantly associated with an increased risk of high TG, high TC, high LDL-C, and CCRFs, with ORs of 1.172, 1.121, 1.169, and 1.175, respectively. After further adjustment for BMI, those correlations continued to be significant **(Figure S5g-S5h)**. Pancreatic FF was significantly associated with increased risk of hypertension, T2DM, high TG, low HDL-C, and CCRFs, with ORs of 1.104, 1.082, 1.060, 1.061, and 1.086, respectively. After further adjustment for BMI, those correlations continued to be significant **(Figure S5i-S5j)**. Although SAT area, SAT FF and pPAT FF was significantly associated with increased risk of several dichotomous cardiometabolic risk factors. After further adjustment for BMI, only few correlations remianed significant. In addition, intramuscular FF and pPAT area were not significantly associated with increased risk of any dichotomous cardiometabolic risk factors **(Table S5**). The significant relationships between various MRI-acquired fat measurements and dichotomous cardiometabolic risk factors independent of BMI in men and women were shown in **Figure S6.**

***Figure S1:*** Association of pPAT area with diastolic BP, FPG, TG, and HDL-C (a) and further adjustment for BMI (b) in men expressed by β coefficients and 95% confidence intervals. Higher pPAT area was associated with higher diastolic BP, higher FPG, higher TG, and lower HDL-C (β = 1.045, 0.275, 0.121, and -0.035, respectively) (a); after further adjustment for BMI, all of those correlations continued to be significant except for diastolic BP and FPG(b).

***Figure S2:*** Association of VAT area (a), VAT FF (c), V/S (e), and hepatic FF (g) with continuous cardiometabolic risk factors and further adjustment for BMI (b, d, f, and h) in women expressed by β coefficients and 95% confidence intervals. Higher VAT area was associated with higher FPG, higher TG, higher TC, lower HDL-C, and higher LDL-C (a). After further adjustment for BMI, those correlations continued to be significant (b). Higher VAT FF was associated with higher FPG, higher TG, higher TC, lower HDL-C, and higher LDL-C (c). After further adjustment for BMI, those correlations continued to be significant, except for higher FPG (d). Higher V/S was associated with higher systolic BP, higher diastolic BP, higher TG, higher TC, and higher LDL-C (e). After further adjustment for BMI, those correlations continued to be significant, except for higher systolic BP (f). Higher hepatic FF was associated with higher FPG, higher TG, higher TC, and higher LDL-C (g). After further adjustment for BMI, those correlations continued to be significant (h).

***Figure S3:*** Graphical summary of the important correlates between various MRI-acquired fat measurements and continuous cardiometabolic risk factors independent of BMI shown in men (a) and women (b) in the present study.

***Figure S4:*** Association of VAT area (a), V/S (c), pancreatic FF (e), and pPAT area (f) with dichotomous cardiometabolic risk factors and further adjustment for BMI (b, d, f, and h) in men expressed by odds ratios and 95% confidence intervals. VAT area was significantly associated with increased risk of hypertension, T2DM, high TG, low HDL-C, and CCRFs (a). After further adjustment for BMI, those correlations continued to be significant (b). V/S was significantly associated with increased risk of hypertension, T2DM, low HDL-C, and CCRFs (c), respectively. After further adjustment for BMI, those correlations continued to be significant (d). Hepatic FF was significantly associated with increased risk of high TG and low HDL-C, with ORs of 1.126 and 1.144, respectively. After further adjustment for BMI, those correlations continued to be significant. Pancreatic FF was significantly associated with increased risk of T2DM, high TG, low HDL-C, and CCRFs (e). After further adjustment for BMI, those correlations continued to be significant (f). pPAT area was significantly associated with increased risk of hypertension, T2DM, high TG, low HDL-C, and CCRFs (g). After further adjustment for BMI, those correlations continued to be significant (h).

***Figure S5:*** Association of VAT area (a), VAT FF (c), V/S (e), hepatic FF (g), and pancreatic FF (i) with dichotomous cardiometabolic risk factors and further adjustment for BMI (b, d, f, h and j) in women are expressed by odds ratios and 95% confidence intervals. VAT area was significantly associated with increased risk of hypertension, T2DM, high TG, high TC, high LDL-C, and CCRFs (a). After further adjustment for BMI, those correlations continued to be significant (b). VAT FF was significantly associated with increased risk of hypertension, high TG, high TC, low HDL-C, high LDL-C, and CCRFs (c). After further adjustment for BMI, those correlations continued to be significant (d). V/S was significantly associated with increased risk of hypertension, T2DM, high TG, high TC, high LDL-C, and CCRFs (e). After further adjustment for BMI, those correlations continued to be significant (f). Hepatic FF was significantly associated with an increased risk of high TG, high TC, high LDL-C, and CCRFs (g). After further adjustment for BMI, those correlations continued to be significant (h). Pancreatic FF was significantly associated with increased risk of hypertension, T2DM, high TG, low HDL-C, and CCRFs (i). After further adjustment for BMI, those correlations continued to be significant (j).

***Figure S6:*** Graphical summary of the important correlates between various MRI-acquired fat measurements and dichotomous cardiometabolic risk factors independent of BMI in men (a) and women (b) shown in the present study.

**Table S1.** Two-observer measurement consistency

| MRI-acquired fat measurements | Radiologist A1 | Radiologist A2 | ICC value  (Intra-observer) | Radiologist B | ICC value  (Inter-observer) |
| --- | --- | --- | --- | --- | --- |
| SAT area (cm^2^) | 133.93 ± 49.04 | 133.78 ± 49.14 | 0.999 | 134.17 ± 48.06 | 0.999 |
| SAT FF (%) | 81.02 ± 4.48 | 81.17 ± 5.40 | 0.950 | 81.22 ± 4.99 | 0.946 |
| VAT area (cm^2^) | 149.13 ± 75.51 | 148.95 ± 75.58 | 0.999 | 150.22 ± 76.30 | 0.999 |
| VAT FF (%) | 78.31 (74.51, 84.32) | 77.88 (75.55, 81.24) | 0.948 | 78.67 (74.88, 80.99) | 0.946 |
| V/S | 0.91 (0.69, 1.66) | 1.13 ± 0.59 | 0.998 | 1.14 ± 0.60 | 0.999 |
| Hepatic FF (%) | 4.00 (2.90, 6.20) | 3.95 (2.90, 5.90) | 0.997 | 4.00 (3.00, 6.40) | 0.998 |
| Pancreatic FF (%) | 8.50 (5.30, 12.30) | 8.00 (5.20, 12.30) | 0.995 | 8.70 (5.30, 12.10) | 0.998 |
| Intramuscular FF (%) | 4.75 (3.80, 7.20) | 5.00 (3.50, 7.20) | 0.987 | 5.00 (3.50, 7.40) | 0.992 |
| pPAT area (cm^2^) | 3.35 ± 1.68 | 3.35 ± 1.67 | 0.993 | 3.33 ± 1.65 | 0.998 |
| pPAT FF (%) | 84.65 (77.90, 87.70) | 84.95 (78.40, 86.60) | 0.998 | 85.25 (77.20, 87.50) | 0.998 |

Data are mean ± SD, median (25, 75 percentile).

ICC, the intraclass correlation coefficient; SAT, subcutaneous adipose tissue; FF, fat fraction; VAT, visceral adipose tissue; V/S, visceral/subcutaneous adipose tissue area ratio.

**Table S2.** The age-adjusted sex-specific correlations among MRI-acquired fat measurements

| MRI-acquired fat measurements | SAT area | SAT FF | VAT area | VAT FF | V/S | Hepatic FF | Pancreatic FF | Intramuscular FF | pPAT area | pPAT FF |
| --- | --- | --- | --- | --- | --- | --- | --- | --- | --- | --- |
| Men |  |  |  |  |  |  |  |  |  |  |
| SAT area | — | 0.743** | 0.255* | -0.106 | -0.237 | 0.159 | 0.187* | 0.159 | 0.562** | 0.206* |
| SAT FF | 0.743** | — | 0.267* | -0.159 | -0.193* | 0.253* | 0.182* | 0.133 | 0.417** | 0.307** |
| VAT area | 0.255* | 0.267* | — | -0.074 | 0.806** | 0.259* | 0.394* | 0.065 | 0.396** | 0.166* |
| VAT FF | -0.106 | -0.159 | -0.074 | — | 0.046 | -0.043 | -0.085 | -0.045 | -0.042 | 0.048 |
| V/S | -0.237* | -0.193* | 0.806* | 0.046 | — | 0.065 | 0.170* | -0.030 | 0.040 | 0.040 |
| Hepatic FF | 0.159 | 0.253* | 0.259* | -0.043 | 0.065 | — | 0.157 | 0.069 | 0.247* | 0.177* |
| Pancreatic FF | 0.187* | 0.182* | 0.394** | -0.085 | 0.170* | 0.157 | — | 0.162 | 0.257* | 0.161 |
| Intramuscular FF | 0.159 | 0.133 | 0.065 | -0.045 | -0.030 | 0.069 | 0.162 | — | -0.024 | 0.020 |
| pPAT area | 0.562** | 0.417** | 0.396** | 0.042 | 0.040 | 0.247* | 0.257* | -0.024 | — | 0.183* |
| pPAT FF | 0.206* | 0.307** | 0.166* | 0.048 | 0.040 | 0.177* | 0.161* | 0.020 | 0.183* | — |
| Women |  |  |  |  |  |  |  |  |  |  |
| SAT area | — | 0.593** | 0.509** | 0.621** | -0.039 | 0.369** | 0.370** | 0.315** | 0.074 | 0.116 |
| SAT FF | 0.593** | — | 0.371** | 0.625** | 0.081 | 0.228* | 0.238** | 0.025 | 0.032 | 0.258* |
| VAT area | 0.509** | 0.371** | — | 0.634** | 0.821** | 0.371** | 0.350** | 0.207* | 0.034 | 0.209* |
| VAT FF | 0.621** | 0.625** | 0.634** | — | 0.326** | 0.431** | 0.379** | 0.204* | 0.006 | 0.316** |
| V/S | -0.039 | 0.081 | 0.821** | 0.326** | — | 0.144 | 0.118 | 0.024 | -0.015 | 0.135 |
| Hepatic FF | 0.369** | 0.228* | 0.371** | 0.431** | 0.144 | — | 0.250* | 0.124 | 0.028 | 0.184* |
| Pancreatic FF | 0.370** | 0.238** | 0.350** | 0.379** | 0.118 | 0.250** | — | 0.191* | 0.035 | 0.151* |
| Intramuscular FF | 0.315** | 0.025 | 0.207* | 0.204* | 0.024 | 0.124 | 0.191* | — | 0.102 | 0.079 |
| pPAT area | 0.074 | 0.032 | 0.034 | 0.006 | -0.015 | 0.028 | 0.035 | 0.102 | — | -0.015 |
| pPAT FF | 0.116 | 0.258* | 0.209* | 0.316** | 0.135 | 0.184* | 0.151* | 0.079 | -0.015 | — |

*P<0.05, **P<0.001.

BMI, body mass index; SAT, subcutaneous adipose tissue; FF, fat fraction; VAT, visceral adipose tissue; V/S, visceral/subcutaneous adipose tissue area ratio; pPAT, preperitoneal adipose tissue.

**Table S3.** The age-adjusted sex-specific correlations between fat measurements and continuous cardiometabolic risk factors

| MRI-acquired fat measurements | Continuous cardiometabolic risk factors | | | | | | |
| --- | --- | --- | --- | --- | --- | --- | --- |
|  | Systolic BP | Diastolic BP | FPG | TG | TC | HDL-C | LDL-C |
| Men | | | | | | | |
| SAT area | 0.190* | 0.349** | 0.121 | 0.077 | 0.007 | -0.156 | 0.048 |
| SAT FF | -0.033 | 0.206* | 0.079 | 0.063 | 0.042 | -0.066 | 0.067 |
| VAT area | 0.107 | 0.214* | 0.343** | 0.124 | 0.037 | -0.247* | 0.085 |
| VAT FF | 0.039 | 0.009 | -0.062 | -0.026 | -0.140 | -0.102 | -0.114 |
| V/S | 0.073 | 0.042 | 0.265* | 0.032 | -0.004 | -0.144 | 0.021 |
| Hepatic FF | 0.068 | 0.162 | 0.167* | 0.240* | 0.041 | -0.066 | 0.058 |
| Pancreatic FF | 0.081 | 0.077 | 0.258* | 0.167* | 0.030 | -0.233* | 0.047 |
| Intramuscular FF | 0.052 | 0.109 | -0.044 | 0.018 | -0.029 | -0.073 | -0.021 |
| pPAT area | 0.193* | 0.294** | 0.308** | 0.190* | 0.025 | -0.190* | 0.065 |
| pPAT FF | 0.152 | 0.174* | 0.125 | 0.102 | 0.027 | -0.061 | 0.009 |
| Women | | | | | | | |
| SAT area | 0.056 | -0.017 | 0.134 | 0.263* | 0.018 | -0.237* | 0.032 |
| SAT FF | -0.007 | -0.046 | 0.094 | 0.152* | -0.023 | -0.181* | 0.013 |
| VAT area | 0.198* | 0.201* | 0.235* | 0.337** | 0.197* | -0.145 | 0.212* |
| VAT FF | 0.191* | 0.141 | 0.198* | 0.454** | 0.192* | -0.270** | 0.247* |
| V/S | 0.173* | 0.217* | 0.159* | 0.201* | 0.206* | -0.005 | 0.216* |
| Hepatic FF | 0.103 | 0.112 | 0.175* | 0.378** | 0.184* | -0.138 | 0.191* |
| Pancreatic FF | 0.086 | 0.113 | 0.235* | 0.205* | 0.059 | -0.169* | 0.091 |
| Intramuscular FF | 0.188* | 0.056 | 0.008 | 0.041 | 0.054 | -0.075 | 0.072 |
| pPAT area | 0.039 | 0.003 | 0.063 | 0.074 | 0.042 | 0.065 | 0.041 |
| pPAT FF | 0.039 | 0.074 | 0.123 | 0.250* | 0.041 | -0.127 | 0.032 |

*P<0.05, **P<0.001.

BMI, body mass index; SAT, subcutaneous adipose tissue; FF, fat fraction; VAT, visceral adipose tissue; V/S, visceral/subcutaneous adipose tissue area ratio. pPAT, preperitoneal adipose tissue. BP, blood pressure; FPG, fasting plasma glucose; TG, triglycerides; TC, total cholesterol; HDL-C, high-density lipoprotein cholesterol; LDL-C, low-density lipoprotein cholesterol.

**Table S4.** Correlations between continuous cardiometabolic risk factors and MRI-acquired fat measurements stratified by sex

| Risk factors | Model Type ^a^ | Effect Size | SAT area | SAT FF | VAT area | VAT FF | V/S | Hepatic FF | Pancreatic FF | Intramuscular FF | pPAT area | pPAT FF |
| --- | --- | --- | --- | --- | --- | --- | --- | --- | --- | --- | --- | --- |
| Men |  |  |  |  |  |  |  |  |  |  |  |  |
| Systolic BP ^b^ | MV | β (95%CI) | / | / | / | / | / | / | / | / | / | / |
|  |  | P-value | / | / | / | / | / | / | / | / | / | / |
|  | MV+BMI | β (95%CI) | / | / | / | / | / | / | / | / | / | / |
|  |  | P-value | / | / | / | / | / | / | / | / | / | / |
| Diastolic BP ^b^ | MV | β (95%CI) | 0.068 (0.035, 0.101) | 0.303 (0.046, 0.561) | 0.016 (0.000, 0.032) | / | / | / | / | / | 1.045 (0.279, 1.811) | / |
|  |  | P-value | < 0.001 | 0.021 | 0.047 | / | / | / | / | / | 0.008 | / |
|  | MV+BMI | β (95%CI) | 0.068 (0.035, 0.101) | / | / | / | / | / | / | / | / | / |
|  |  | P-value | < 0.001 | / | / | / | / | / | / | / | / | / |
| FPG ^c^ | MV | β (95%CI) | / | / | 0.005 (0.002, 0.008) | / | 0.182 (0.004, 0.361) | 0.082 (0.021, 0.143) | 0.036 (0.005, 0.068) | / | 0.275 (0.151, 0.398) | / |
|  |  | P-value | / | / | < 0.001 | / | 0.045 | 0.009 | 0.005 | / | < 0.001 | / |
|  | MV+BMI | β (95%CI) | / | / | / | / | / | / | / | / | / | / |
|  |  | P-value | / | / | / | / | / | / | / | / | / | / |
| TG ^d^ | MV | β (95%CI) | / | / | / | / | / | 0.077 (0.028, 0.125) | / | / | 0.121 (0.017, 0.226) | / |
|  |  | P-value | / | / | / | / | / | 0.002 | / | / | 0.023 | / |
|  | MV+BMI | β (95%CI) | / | / | / | / | / | 0.077 (0.028, 0.125) | / | / | 0.121 (0.017, 0.226) | / |
|  |  | P-value | / | / | / | / | / | 0.002 | / | / | 0.023 | / |
| TC ^d^ | MV | β (95%CI) | / | / | / | / | / | / | / | / | / | / |
|  |  | P-value | / | / | / | / | / | / | / | / | / | / |
|  | MV+BMI | β (95%CI) | / | / | / | / | / | / | / | / | / | / |
|  |  | P-value | / | / | / | / | / | / | / | / | / | / |
| HDL-C ^d^ | MV | β (95%CI) | -0.002 (-0.003, 0.000) | / | -0.001(-0.001,0.000) | / | / | / | -0.010 (-0.017, -0.003) | / | -0.035 (-0.064, -0.006) | / |
|  |  | P-value | 0.025 | / | 0.003 | / | / | / | 0.004 | / | 0.018 | / |
|  | MV+BMI | β (95%CI) | -0.002 (-0.003, 0.000) | / | -0.001(-0.001,0.000) | / | / | / | -0.010 (-0.017, -0.003) | / | -0.035 (-0.064, -0.006) | / |
|  |  | P-value | 0.025 | / | 0.003 | / | / | / | 0.004 | / | 0.018 | / |
| LDL-C ^d^ | MV | β (95%CI) | / | / | / | / | / | / | / | / | / | / |
|  |  | P-value | / | / | / | / | / | / | / | / | / | / |
|  | MV+BMI | β (95%CI) | / | / | / | / | / | / | / | / | / | / |
|  |  | P-value | / | / | / | / | / | / | / | / | / | / |
| Women |  |  |  |  |  |  |  |  |  |  |  |  |
| Systolic BP ^b^ | MV | β (95%CI) | / | / | / | / | 12.167 (5.464, 18.871) | / | / | 0.810 (0.309, 1.312) | / | / |
|  |  | P-value | / | / | / | / | < 0.001 | / | / | 0.002 | / | / |
|  | MV+BMI | β (95%CI) | / | / | / | / | / | / | / | 0.668 (0.153, 1.184) | / | / |
|  |  | P-value | / | / | / | / | / | / | / | 0.011 | / | / |
| Diastolic BP ^b^ | MV | β (95%CI) | / | / | / | / | 7.713 (3.268, 12.159) | / | / | / | / | / |
|  |  | P-value | / | / | / | / | 0.001 | / | / | / | / | / |
|  | MV+BMI | β (95%CI) | / | / | / | / | 7.713 (3.268, 12.159) | / | / | / | / | / |
|  |  | P-value | / | / | / | / | 0.001 | / | / | / | / | / |
| FPG ^c^ | MV | β (95%CI) | 0.005 (0.001, 0.010) | / | 0.008 (0.003, 0.012) | 0.063 (0.014, 0.112) | / | 0.087 (0.027, 0.147) | / | / | / | / |
|  |  | P value | 0.028 | / | < 0.001 | 0.012 | / | 0.005 | / | / | / | / |
|  | MV+BMI | β (95%CI) | / | / | 0.008 (0.003, 0.012) | / | / | 0.087 (0.027, 0.147) | / | / | / | / |
|  |  | P-value | / | / | < 0.001 | / | / | 0.005 | / | / | / | / |
| TG ^d^ | MV | β (95%CI) | 0.004 (0.002, 0.007) | / | 0.007 (0.005,0.009) | 0.081 (0.057, 0.105) | 0.984 (0.599, 1.369) | 0.083 (0.052, 0.114) | 0.023 (0.007, 0.039) | / | / | 0.019 (0.008, 0.030) |
|  |  | P-value | < 0.001 | / | < 0.001 | < 0.001 | < 0.001 | < 0.001 | 0.005 | / | / | 0.001 |
|  | MV+BMI | β (95%CI) | / | / | 0.007 (0.005,0.009) | 0.081 (0.057, 0.105) | 0.836 (0.435, 1.238) | 0.071 (0.039, 0.104) | / | / | / | 0.015 (0.004, 0.027) |
|  |  | P-value | / | / | < 0.001 | < 0.001 | 0.024 | < 0.001 | / | / | / | 0.008 |
| TC ^d^ | MV | β (95%CI) | / | / | 0.004 (0.001, 0.006) | 0.051 (0.012, 0.090) | 1.045 (0.431, 1.658) | 0.059 (0.011,0.106) | / | / | / | / |
|  |  | P-value | / | / | 0.010 | 0.011 | 0.001 | 0.015 | / | / | / | / |
|  | MV+BMI | β (95%CI) | / | / | 0.004 (0.001, 0.006) | 0.051 (0.012, 0.090) | 1.045 (0.431, 1.658) | 0.059 (0.011,0.106) | / | / | / | / |
|  |  | P-value | / | / | 0.010 | 0.011 | 0.001 | 0.015 | / | / | / | / |
| HDL-C ^d^ | MV | β (95%CI) | -0.002 (-0.003, -0.001) | -0.022 (-0.038, -0.006) | -0.002 (-0.003, -0.001) | -0.027 (-0.040, -0.014) | / | / | -0.009 (-0.017, -0.001) | / | / | / |
|  |  | P-value | 0.002 | 0.009 | < 0.001 | <0.001 | / | / | 0.024 | / | / | / |
|  | MV+BMI | β (95%CI) | -0.002 (-0.003, -0.001) | -0.022 (-0.038, -0.006) | -0.002 (-0.003, -0.001) | -0.027 (-0.040, -0.014) | / | / | -0.009 (-0.017, -0.001) | / | / | / |
|  |  | P-value | 0.002 | 0.009 | < 0.001 | <0.001 | / | / | 0.024 | / | / | / |
| LDL-C ^d^ | MV | β (95%CI) | / | / | 0.005 (0.003, 0.008) | 0.058 (0.027, 0.088) | 1.126 (0.671, 1.581) | 0.049 (0.011, 0.087) | / | / | / | / |
|  |  | P-value | / | / | 0.009 | < 0.001 | < 0.001 | 0.012 | / | / | / | / |
|  | MV+BMI | β (95%CI) | / | / | 0.005 (0.003, 0.008) | 0.058 (0.027, 0.088) | 1.126 (0.671, 1.581) | 0.049 (0.011, 0.087) | / | / | / | / |
|  |  | P-value | / | / | 0.009 | < 0.001 | < 0.001 | 0.012 | / | / | / | / |

Blank cells indicate that the MRI-acquired fat depots were not selected using the forward selection regression procedure.

^a^ MV model included the following covariates: age, smoking status, current alcohol use, family history of cardiometabolic risk factors, and postmenopausal status (women only). BMI was additionally adjusted for in the models labeled “MV+BMI model”.

^b^ Antihypertensive treatment was included as a covariate for systolic and diastolic blood pressure models.

^c^ Diabetes treatment was included as a covariate for the fasting plasma glucose model.

^d^ Lipid-lowering treatment was included as a covariate for TG and HDL-C models.

BMI, body mass index; SAT, subcutaneous adipose tissue; FF, fat fraction; VAT, visceral adipose tissue; V/S, visceral/subcutaneous adipose tissue area ratio. pPAT, preperitoneal adipose tissue. BP, blood pressure; FPG, fasting plasma glucose; TG, triglycerides; TC, total cholesterol; HDL-C, high-density lipoprotein cholesterol; LDL-C, low-density lipoprotein cholesterol.

**Table S5.** Correlations between dichotomous cardiometabolic risk factors and MRI-acquired fat measurements stratified by sex

| Risk factors | Model Type ^a^ | Effect Size | SAT area | SAT FF | VAT area | VAT FF | V/S | Hepatic FF | Pancreatic FF | Intramuscular FF | pPAT area | pPAT FF |
| --- | --- | --- | --- | --- | --- | --- | --- | --- | --- | --- | --- | --- |
| Men |  |  |  |  |  |  |  |  |  |  |  |  |
| Hypertension | MV | OR  (95%CI) | 1.011 (1.002, 1.021) | / | 1.009 (1.003, 1.014) | / | 1.451 (0.898, 2.345) | / | / | / | 1.496 (1.192, 1.877) | / |
|  |  | P-value | 0.020 | / | 0.002 | / | 0.128 | / | / | / | 0.001 | / |
|  | MV+BMI | OR  (95%CI) | / | / | 1.005 (1.000, 1.010) | / | 1.310 (0.890, 1.930) | / | / | / | 1.275 (0.980, 1.658) | / |
|  |  | P-value | / | / | 0.068 | / | 0.171 | / | / | / | 0.070 | / |
| T2DM | MV | OR  (95%CI) | / | / | 1.007 (1.002, 1.012) | / | 1.888 (1.068, 3.336) | / | 1.102 (1.050, 1.158) | / | 1.262 (1.029, 1.548) | / |
|  |  | P-value | / | / | 0.010 | / | 0.029 | / | <0.001 | / | 0.026 | / |
|  | MV+BMI | OR  (95%CI) | / | / | 1.007 (1.002, 1.012) | / | 1.888 (1.068, 3.336) | / | 1.102 (1.050, 1.158) | / | 1.262 (1.029, 1.548) | / |
|  |  | P-value | / | / | 0.010 | / | 0.029 | / | <0.001 | / | 0.026 | / |
| High TG | MV | OR  (95%CI) | / | / | 1.006 (1.001, 1.011) | / | / | 1.126 (1.022, 1.240) | 1.075 (1.024, 1.129) | / | 1.254 (1.015, 1.548) | / |
|  |  | P-value | / | / | 0.022 | / | / | 0.016 | 0.003 | / | 0.036 | / |
|  | MV+BMI | OR  (95%CI) | / | / | 1.006 (1.001, 1.011) | / | / | 1.126 (1.022, 1.240) | 1.075 (1.024, 1.129) | / | 1.254 (1.015, 1.548) | / |
|  |  | P-value | / | / | 0.022 | / | / | 0.016 | 0.003 | / | 0.036 | / |
| High TC | MV | OR  (95%CI) | / | / | / | / | / | / | / | / | / | / |
|  |  | P-value | / | / | / | / | / | / | / | / | / | / |
|  | MV+BMI | OR  (95%CI) | / | / | / | / | / | / | / | / | / | / |
|  |  | P-value |  | / | / | / | / | / | / | / | / | / |
| Low HDL-C | MV | OR  (95%CI) | / | / | 1.011 (1.005, 1.017) | / | 2.034 (1.236, 3.346) | 1.144 (1.032, 1.269) | 1.060 (1.014, 1.108) | / | 1.404 (1.140, 1.729) | / |
|  |  | P-value | / | / | <0.001 | / | 0.005 | 0.011 | 0.011 | / | 0.001 | / |
|  | MV+BMI | OR  (95%CI) | / | / | 1.011 (1.005, 1.017) | / | 2.034 (1.236, 3.346) | 1.144 (1.032, 1.269) | 1.060 (1.014, 1.108) | / | 1.404 (1.140, 1.729) | / |
|  |  | P-value | / | / | <0.001 | / | 0.005 | 0.011 | 0.011 | / | 0.001 | / |
| High LDL-C | MV | OR  (95%CI) | / | / | / | / | / | / | / | / | / | / |
|  |  | P-value | / | / | / | / | / | / | / | / | / | / |
|  | MV+BMI | OR  (95%CI) | / | / | / | / | / | / | / | / | / | / |
|  |  | P-value | / | / | / | / | / | / | / | / | / | / |
| CCRFs | MV | OR  (95%CI) | / | / | 1.011 (1.006, 1.017) | / | 2.437 (1.458, 4.074) | / | 1.090 (1.038, 1.143) | / | 1.288 (1.060, 1.566) | 1.067 (1.012, 1.125) |
|  |  | P-value | / | / | <0.001 | / | 0.001 | / | 0.001 | / | 0.011 | 0.016 |
|  | MV+BMI | OR  (95%CI) | / | / | 1.011 (1.006, 1.017) | / | 2.392 (1.414, 4.045) | / | 1.090 (1.038, 1.143) | / | 1.288 (1.060, 1.566) | 1.067 (1.012, 1.125) |
|  |  | P-value | / | / | <0.001 | / | 0.001 | / | 0.001 | / | 0.011 | 0.016 |
| Women |  |  |  |  |  |  |  |  |  |  |  |  |
| Hypertension | MV | OR  (95%CI) | 1.007 (1.001, 1.014) | / | 1.013 (1.006, 1.021) | 1.175 (1.069, 1.291) | 5.470 (1.662, 18.001) | / | 1.104 (1.036, 1.176) | / | / | / |
|  |  | P-value | 0.032 | / | < 0.001 | 0.001 | 0.005 | / | 0.002 | / | / | / |
|  | MV+BMI | OR  (95%CI) | / | / | 1.013 (1.006, 1.021) | 1.175 (1.069, 1.291) | 4.054 (1.610, 14.150) | / | 1.104 (1.036, 1.176) | / | / | / |
|  |  | P-value | / | / | < 0.001 | 0.001 | 0.028 | / | 0.002 | / | / | / |
| T2DM | MV | OR  (95%CI) | / | / | 1.012 (1.004, 1.020) | / | 5.159 (1.330, 20.009) | / | 1.082 (1.030, 1.136) | / | / | / |
|  |  | P-value | / | / | 0.005 | / | 0.018 | / | 0.002 | / | / | / |
|  | MV+BMI | OR  (95%CI) | / | / | 1.012 (1.004, 1.020) | / | 5.159 (1.330, 20.009) | / | 1.082 (1.030, 1.136) | / | / | / |
|  |  | P-value | / | / | 0.005 | / | 0.018 | / | 0.002 | / | / | / |
| High TG | MV | OR  (95%CI) | 1.009 (1.002, 1.016) | / | 1.016 (1.008, 1.023) | 1.250 (1.123, 1.391) | 8.559 (2.595,28.226) | 1.172 (1.072, 1.281) | 1.060 (1.015, 1.107) | / | / | 1.121 (1.045, 1.203) |
|  |  | P-value | 0.013 | / | <0.001 | <0.001 | <0.001 | <0.001 | 0.009 | / | / | 0.001 |
|  | MV+BMI | OR  (95%CI) | / | / | 1.016 (1.008, 1.023) | 1.250 (1.121, 1.391) | 6.327 (1.786, 22.408) | 1.136 (1.036, 1.246) | 1.042 (0.998, 1.087) | / | / | / |
|  |  | P-value | / | / | <0.001 | <0.001 | 0.004 | 0.007 | 0.061 | / | / | / |
| High TC | MV | OR  (95%CI) | / | / | 1.013 (1.005, 1.020) | 1.181 (1.067, 1.308) | 19.919 (4.901, 80.964) | 1.121 (1.025, 1.225) | / | / | / | / |
|  |  | P-value | / | / | 0.001 | 0.001 | <0.001 | 0.012 | / | / | / | / |
|  | MV+BMI | OR  (95%CI) | / | / | 1.013 (1.005, 1.020) | 1.181 (1.067, 1.308) | 19.919 (4.901, 80.964) | 1.121 (1.025, 1.225) | / | / | / | / |
|  |  | P-value | / | / | 0.001 | 0.001 | <0.001 | 0.012 | / | / | / | / |
| Low HDL-C | MV | OR  (95%CI) | 1.011 (1.004, 1.019) | 1.228 (1.074, 1.403) | / | 1.150 (1.047, 1.262) | / | / | 1.061 (1.014, 1.110) | / | / | 1.094 (1.021, 1.171) |
|  |  | P-value | 0.004 | 0.003 | / | 0.003 | / | / | 0.010 | / | / | 0.010 |
|  | MV+BMI | OR  (95%CI) | 1.011 (1.004, 1.019) | 1.228 (1.074, 1.403) | / | 1.150 (1.047, 1.262) | / | / | 1.061 (1.014, 1.110) | / | / | 1.094 (1.021, 1.171) |
|  |  | P-value | 0.004 | 0.003 | / | 0.003 | / | / | 0.010 | / | / | 0.010 |
| High LDL-C | MV | OR  (95%CI) | / | / | 1.013 (1.004, 1.022) | 1.134 (0.997, 1.291) | 26.457 (5.193, 134.781) | 1.169 (1.052, 1.298) | / | / | / | / |
|  |  | P-value | / | / | 0.004 | 0.056 | <0.001 | 0.004 | / | / | / | / |
|  | MV+BMI | OR  (95%CI) | / | / | 1.013 (1.004, 1.022) | 1.134 (0.997, 1.291) | 26.457 (5.193, 134.781) | 1.169 (1.052, 1.298) | / | / | / | / |
|  |  | P-value | / | / | 0.004 | 0.056 | <0.001 | 0.004 | / | / | / | / |
| CCRFs | MV | OR  (95%CI) | 1.009 (1.002, 1.016) | / | 1.017 (1.010, 1.025) | 1.221 (1.109, 1.344) | 9.404 (2.821, 31.353) | 1.175 (1.071, 1.289) | 1.086 (1.029, 1.147) | / | / | 1.067 (1.011, 1.127) |
|  |  | P-value | 0.008 | / | <0.001 | <0.001 | <0.001 | 0.001 | 0.003 | / | / | 0.019 |
|  | MV+BMI | OR  (95%CI) | / | / | 1.017 (1.010, 1.025) | 1.221 (1.109, 1.344) | 7.122 (2.011, 25.215) | 1.136 (1.033, 1.250) | 1.062 (1.009, 1.118) | / | / | / |
|  |  | P-value | / | / | <0.001 | <0.001 | 0.002 | 0.009 | 0.021 | / | / | / |

Blank cells indicate that the MRI-acquired fat depots were not selected using the forward: LR selection regression procedure.

^a^ MV model included the following covariates: age, smoking status, current alcohol use, family history of cardiometabolic risk factors, and postmenopausal status (women only). BMI was additionally adjusted for in the models labeled “MV+BMI model”.

BMI, body mass index; SAT, subcutaneous adipose tissue; FF, fat fraction; VAT, visceral adipose tissue; V/S, visceral/subcutaneous adipose tissue area ratio. pPAT, preperitoneal adipose tissue; BP, blood pressure; FPG, fasting plasma glucose; TG, triglycerides; TC, total cholesterol; HDL-C, high-density lipoprotein cholesterol; LDL-C, low-density lipoprotein cholesterol; T2DM, type 2 diabetes mellitus; CCRFs, clustering of cardiometabolic risk factors.
